# Comparative Analysis of TM and Cytoplasmic β-barrel Conformations Using Joint Descriptor

**DOI:** 10.1038/s41598-018-32136-4

**Published:** 2018-09-21

**Authors:** Jayaraman Thangappan, Sangwook Wu, Sun-Gu Lee

**Affiliations:** 1 Department of Physics, Pukyong National University, Busan, 608-737 Republic of Korea; 2 Department of Chemical Engineering, Pusan National University, Busan, 609-735 Republic of Korea

## Abstract

Macroscopic descriptors have become valuable as coarse-grained features of complex proteins and are complementary to microscopic descriptors. Proteins macroscopic geometric features provide effective clues in the quantification of distant similarity and close dissimilarity searches for structural comparisons. In this study, we performed a systematic comparison of β-barrels, one of the important classes of protein folds in various transmembrane (TM) proteins against cytoplasmic barrels to estimate the conformational features using a joint-based descriptor. The approach uses joint coordinates and dihedral angles (β and γ) based on the β-strand joints and loops to determine the arrangements and propensities at the local and global levels. We then confirmed that there is a clear preference in the overall β and γ distribution, arrangements of β-strands and loops, signature patterns, and the number of strand effects between TM and cytoplasmic β-barrel geometries. As a robust and simple approach, we determine that the joint-based descriptor could provide a reliable static structural comparison aimed at macroscopic level between complex protein conformations.

## Introduction

β-barrel proteins are among the most significant and abundant entities in both membrane and cytoplasmic proteins as well as helical domains. These β-barrel structures are composed of a repertoire of β-strand segments joined by loops. Each strand is hydrogen bonded to its neighbor in an antiparallel/parallel arrangement, thus forming β sheets. Such β sheets twist up together to form a closed or open cylinder-like structure. Although membrane and cytoplasmic β-barrel proteins differ sequentially in their amino acid propensities, both β-barrel proteins share similar structural folds. In addition, β-barrel proteins can carry out a variety of biological functions, which has gained them wide-spread attention^1–5^. Most transmembrane β-barrel (β-barrels^TM^) structures are closed and act as pores or channels representing uniquely shaped Outer Membrane Proteins (OMP)^6,7^, while the cytoplasmic β-barrel structures (β-barrels^cytoplasm^) are mixed fractions of open and closed structures functioning as catalytic and ligand binding units with diverse topology^8–10^. Most OMPs are detected with an even number of strands ranging from about four to 26^11–14^. On the other hand, most β-barrels^cytoplasm^ proteins are diverse and range between four to 10 strands, with fewer numbers of proteins ranging up to 14 strands^15^. OMP β-barrels are monomeric with a single chain barrel, and barrels of cytoplasmic proteins often show complicated topologies in which more than one barrel contributes to a single chain^16^. Together, both β-barrels^TM^ and β-barrels^cytoplasm^ structures are significant for therapeutic targeting because they resemble the channeling system of helical proteins^17–19^.

While β-barrels^cytoplasm^ structures are clearly determined, β-barrels^TM^ structure determinations are still progressing under ever-increasing challenges in relation to TM protein purification and crystallization^20–22^. Only a few hundred unique β-barrels^TM^ structures have been identified, and these constitute an inconsequential part of the known structure deposited in the Protein Data Bank (PDB), succeeding the membrane helical protein genomes sequenced so far. Supplementary to experimental procedures, computational modeling has been accelerating our understanding of β-barrel proteins^23–25^. However, such analyses have not yet illuminated the nature of β-barrel structures due to the complexities of conformational distribution. Comparison of the intricacies of any distinctive features of β-barrels^TM^ and β-barrels^cytoplasm^ structures could be refined down by simple representations. Several descriptions of protein structures have been illustrated to show the relationship between secondary structural elements (SSEs) and several essential parameters that govern the structure and constraints^26–30^. Elucidation of geometric features using detailed atomic and Cα residue representations is a common approach to the analysis of helical and β-sheet packing characterizations^31–33^. In addition, various levels of coarse-grained models have been suggested to reduce the complexity^34–36^. However, a basic model that relates to the structural arrangement of both β-barrels^TM^ and β-barrels^cytoplasm^ structures at the macroscopic level is required to define the connectivity similarities and dissimilarities. Because β-strand structural arrangements play a critical role in the function and organization of β-barrel proteins, models that utilize the β-strand conformations would be more useful. Furthermore, such model would facilitate the understanding of how β-barrel structural organizations endure both membrane and water-soluble environments while utilizing their β sheet arrangements, overall topology, and conformational heterogeneity. Therefore, methods that identify the macroscopic principles underlying β-barrel protein folding and design factors in the overall structure, function, dynamics, and interactions become promising.

In the present study, we attempted to perform a systematic comparison of β-barrels^TM^ and β-barrels^cytoplasm^ geometries using the joint-based description approach^37,38^. For comparison, structures of high-resolution, non-homologous β-barrels^TM^ and β-barrels^cytoplasm^ protein structures were obtained from the PDB and grouped based on their geometric analyses. We considered the understanding of both β-barrel types with the topological patterns that exhibit diverse structural and functional relationships. We believe that β-barrel structural organizations can be statistically quantified based on β-strands arrangements and conformational distributions. Thus, both β-barrels^TM^ and β-barrels^cytoplasm^ structures were analyzed in terms of two dihedral angles; β and γ. Our results demonstrate that TM β-strands are predominantly right-handed and anti-parallel as reported previously^39,40^, and they are comparatively like the conformational flexibility of TM helices in nature^40–43^. In contrast, structures of β-barrels^cytoplasm^ are short, mixed and varied in their arrangements with one another. All the observed conformational distributions and adjacent frequencies were associated with the arrangement of β-strands and loops in the context of how β-barrel structures have adapted in both membrane and water-soluble environments respectively. Despite uniformity, we observed that there are some considerable differences between β-barrels^TM^ and β-barrels^cytoplasm^ that share an equal number of β-strands. The comparative analysis results of β-barrel proteins prove that the joint-based description approach is a powerful descriptor that can be used to boost the geometrical characterization of β-barrels at both local and global levels. Possibly, our joint-based descriptor approach will be an asset in β-barrels structural prediction, as well as in the in-silico design, modeling, and validation of topologies with the given β-strand and loop segments.

## Results

### Macroscopic descriptor for β-barrel structures

**Figure 1:**
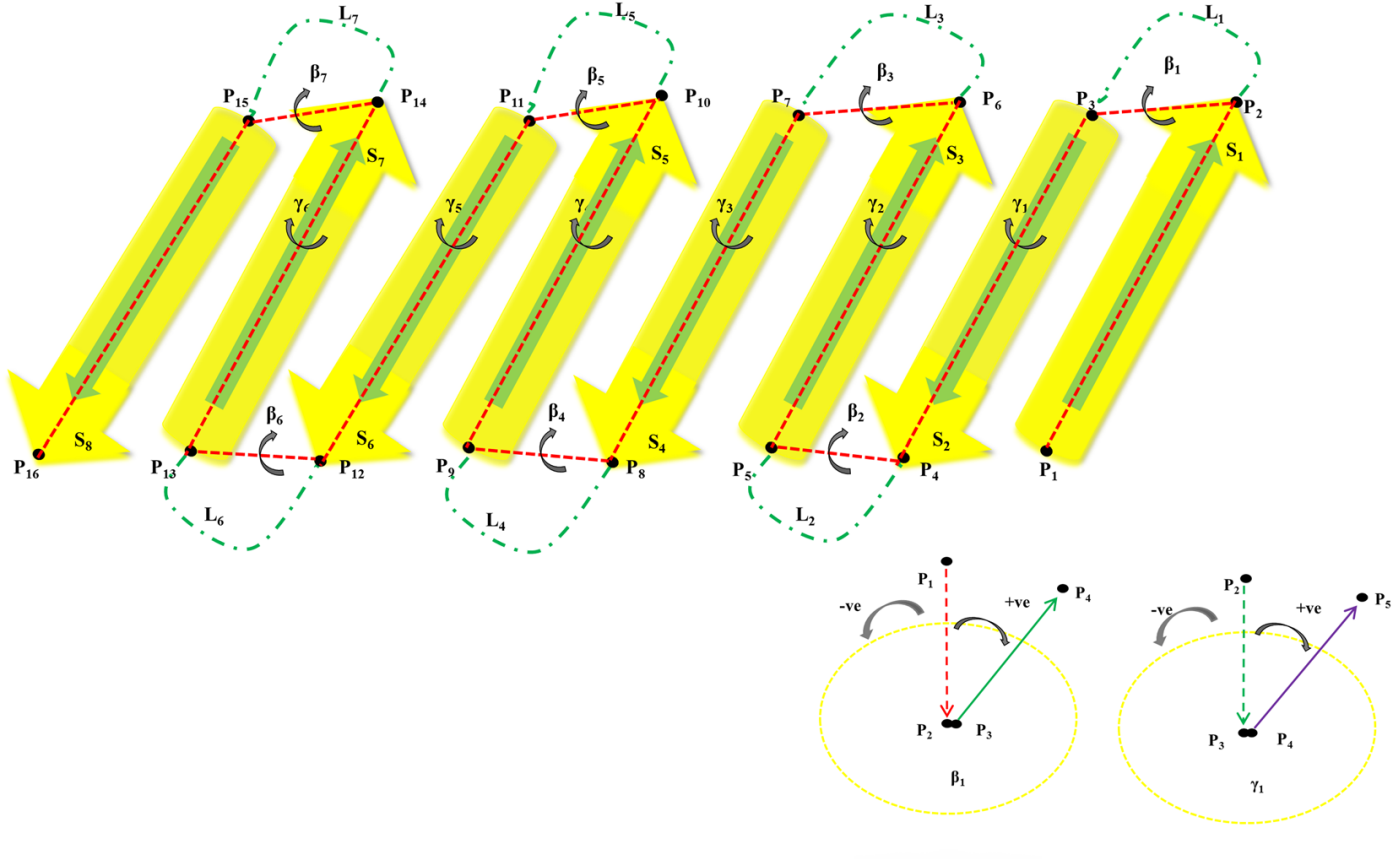
Joint-based description of β-barrel proteins with eight strands and seven loops. (**a**) Assignment of the β type and γ type dihedral angles. S_1_ to S_8_ are β-strands, L_1_ to L_7_ are loops, and P_1_ to P_16_ are joint points. Type β dihedral angles, such as β_1_, are defined by the four joint points in the Strand-Loop-Strand, such as P_1_, P_2_, P_3_, and P_4_. The γ-type dihedral angles are defined by the four joint points in the Loop-Strand-Loop, such as P_2_, P_3_, P_4_, and P_5_. (**b**) Assignment of the positive and negative signs for dihedral angles. The positive (+) sign and negative (−) signs represent the clockwise and counter-clockwise angles, respectively, in the projections for the dihedral angles. The figures present the projections for β_1_ and γ_1_.

The present study deals in relation to the recently developed joint-based description of protein structures. To understand the macroscopic geometric features of both TM and cytoplasmic β-barrel proteins such as their conformational distribution and the arrangements of β-strands, we utilize the joint constraint principles that were previously prepared to represent TM helices^37^. These β-barrel proteins always show consecutive elements of β-strands and loops linked one another as described in Fig. 1. Like TM helical proteins, a set of joints connecting the individual β-strands and loops were selected to describe a β-barrel geometry based on the joint-based description approach. Specifically, the Cα carbon coordinates of the first and last residues of each β-strand were considered as structural joining points as they represent the basic elements of protein structures^37,38^. The spatial arrangement of such joint points was measured by the dihedral angles between them. For example, for a protein composed of eight β-strands (S_1_, S_2_, S_3_, S_4_, S_5_, S_6_, S_7_, and S_8_) and seven loops (L_1_, L_2_, L_3_, L_4_, L_5_, L_6_ and L_7_), a group of 16 joints (P_1_, P_2_, P_3_, P_4_, P_5_, P_6_, P_7_, P_8_, P_9_, P_10_, P_11_, P_12_, P_13_, P_14_, P_15_, and P_16_) can be assigned (Fig. 1). As a result, dihedral angle involving first four joints P_1_, P_2_, P_3_ and P_4_ can be determined by measuring the angle between two planes made by P_1_, P_2_, P_3_ and P_2_, P_3_, P_4_. Then, the second dihedral angle can be found by relating the structural points P_2_, P_3_, P_4_, and P_5_, and P_3_, P_4_, P_5_ and P_6_ joints are used to determine the third, fourth and so on. Here, we propose two new types of dihedral angles: β and γ for β strands and loops. Again, the first and third dihedral angles correspond to the type β. They are denoted as β_1_ and β_2_ respectively. In a similar way, the second and fourth dihedral angles correspond to the type γ, denoted as γ_1_ and γ_2_, respectively. Thus, the β-strand conformations of both β-barrels^TM^ and β-barrels^cytoplasm^ proteins can be represented by the set of joints (P_1_, P_2_, P_3_…) and two types of dihedral angles (β_1_, γ_1_, β_2_, γ_2_, β_3_…) at the macroscopic level. Clockwise and counter-clockwise angle signs were specified based on a positive value (from 0 to 180 degrees) or a negative value (from −180 to 0 degrees) respectively (Fig. 1).

### Dataset collection of targets β-barrel proteins

**Figure 2:**
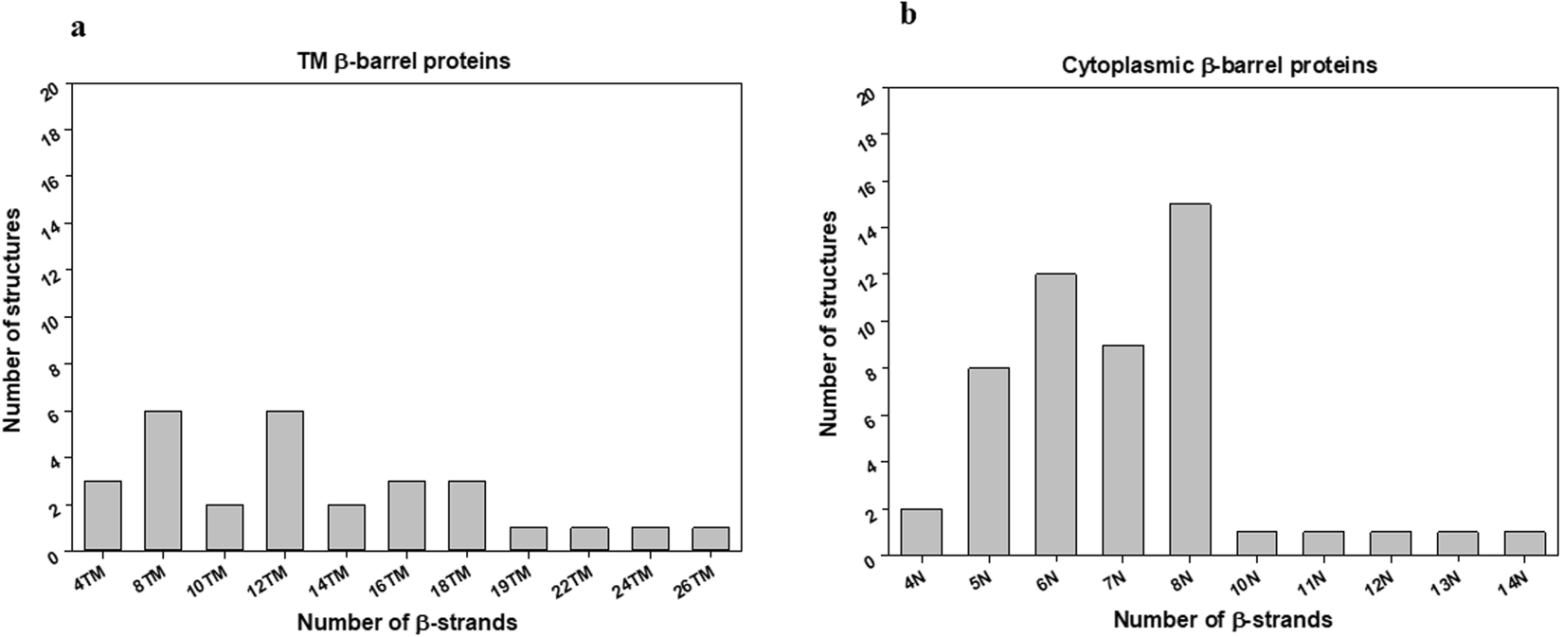
Total number of β-barrel proteins for (**a**) membrane and (**b**) cytoplasmic cases. (**a**) β-barrels^TM^ target dataset of 29 structures was grouped based on the TM β-strand numbers and (**b**) 51 β-barrels^cytoplasm^ structures were also classified based on their β-strand numbers. Note: β-barrels^cytoplasm^ include both closed and open structures.

For β-barrels^TM^, 29 proteins with an even number from 4 to 26 β-strands and odd number 19 TM β-strands protein were collected as an initial dataset. For β-barrels^cytoplasm^, a total of 51 proteins from several superfamilies were chosen. The total number of both TM and cytoplasmic β-barrel proteins with various topologies, β-strands, and loop numbers were listed in Table 1 and Table 2 respectively. These datasets were directly obtained from Protein Data Bank (PDB) by grouping into β-barrels^TM^ and β-barrels^cytoplasm^ proteins. The dataset collection procedure is cited in the *Methods* section. Concisely in both cases, i) We selected 30% sequence criteria and filtered the high-resolution X-ray crystal structures from PDB, ii) We have separated the unique monomer β-barrel proteins, and iii) The non-homologous monomeric chains with respect to different number of β-strand was grouped as final target proteins as shown in Fig. 2(a,b). In the end, the target dataset of 29 and 51 β-barrel structures was analyzed and all such analyzed β and γ angles are grouped and classified (Supporting Information SI Tables (1, 2, 3 and 4)).

**Table 1:** Selected non-homologous β-barrels^TM^ protein structures (29), their PDB IDs, and the total number of β types and γ type dihedral angles used in this study.

Group	PDB IDs	# of Structures	# of β types	# of γ types
4TM	1EK9A, 2GR8A, & 3X2RA	3	9	6
8TM	1P4TA, 1QJPA, 2ERVA, 2X27X, 3DZMA, & 3GP6A	6	30	24
10TM	2VDFA & 2X55A	2	18	16
12TM	1QD6C, 1TLYA, 1UYNX, 2WJRA, 3FIDA, & 4RL8A	6	66	60
14TM	2X9KA, & 3BS0A	2	26	24
16TM	2FGQX, 4C00A, & 4Y25A	3	45	42
18TM	1A0TP, 2YNKA, & 3SZVA	3	51	48
19TM	4C69X	1	18	17
22 TM	1FEPA	1	21	20
24TM	3FIPA	1	23	22
26TM	4Q35A	1	25	24
Total numbers		29	332	329

All protein names and their classifications used in this work are described in the Supplementary Information Table S9.

**Table 2:** Selected non-homologous β-barrels^cytoplasm^ protein structures (51), their PDB IDs, and the total number of β types and γ type dihedral angles used in this study.

Group	PDB IDs	# of Structures	# of β types	# of γ types
4N	2B97A & 1G3PA	2	6	4
5N	1NB9A, 1WHIA, 1PV4A, 1GCPA, 1HK9A, 1R6JA, 1O6AA, & 1G31A	8	32	24
6N	2JDID, 1WJXA, 1AGJA, 1DFUP, 1S98A, 1EFTA, 2D9RA, 2IMLA, 1BCOA, 1TS9A, 2BLNA & 2F1LA	12	60	48
7N	1WPOA, 1IK9A, 1ORUA, 1JEYA, 1RQPA, 1O70A, 1J0WA, 2GUJA, & 1QZ8A	9	54	45
8N	1EAR, 1UE0A, 1A1XA, 1NYCA, 1OEWA, 1XE1A, 1T2WA, 1PQHA, 2CPLA, 1Y12A, 2Q03A, 2F9HA, 1BEBA, 1GQBA, & 1F3UB	15	105	90
10N	2P12A	1	9	8
11N	2FR2A	1	10	9
12N	4FGFA	1	11	10
13N	1NLSA	1	12	11
14N	1H4GA	1	13	12
Total numbers		51	312	261

All protein names and their classifications used in this work are described in the Supplementary Information Table S10.

### Comparison of β and γ distributions and overall arrangements of both β-barrels^TM^ and β-barrels^cytoplasm^

**Figure 3:**
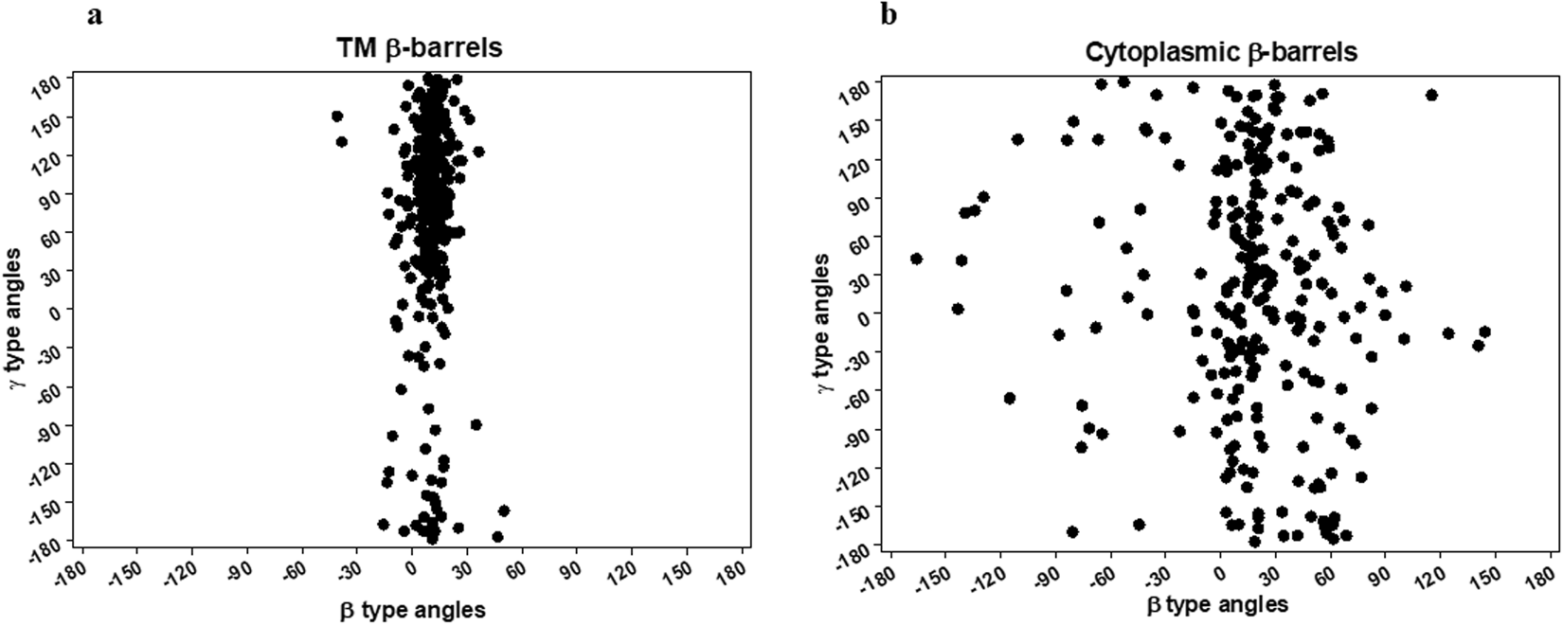
Distribution of the β type and γ type dihedral angles in the β-barrel proteins. (**a**) The β-γ distribution plot for the β-barrels^TM^ proteins. All β and γ type dihedral angles in the 29 non-homologous of β-barrels^TM^ proteins are plotted together in the 2-D scatter plot. (**b**) The β-γ distribution plot for the β-barrels^cytoplasm^ proteins. All β and γ type dihedral angles in the 51 non-homologous of β-barrels^cytoplasm^ proteins are plotted.

**Figure 4:**
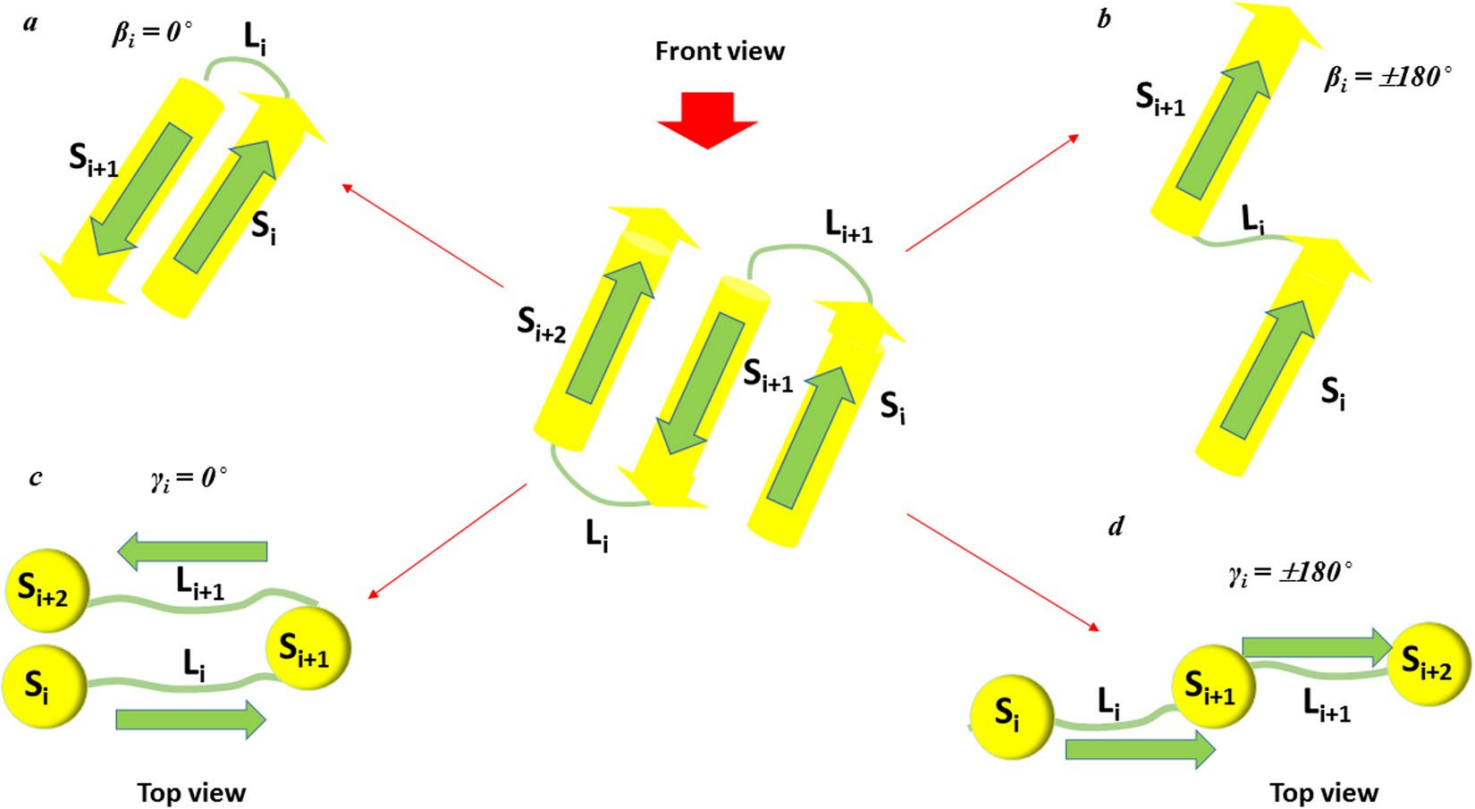
Arrangements of the β-strands and loops depending on the β and γ dihedral angles. The central figure presents the front view of three consecutive β-strands in both β-barrels^TM^ and β-barrels^cytoplasm^ proteins. S_(n)_ represents the β-strands and L_(n)_ represents loops. (**a**) Front view of the arrangement of two β-strands helices, S_i_ and S_i+1_ when β_i_ = 0°, (**b**) Front view of the arrangement of two adjacent helices, S_i_ and S_i+1_ when β_i_ = ±180°, (**c**) Top view of the arrangement of two adjacent loops, L_i_ and L_i+1_, and three adjacent β-strands, S_i_, S_i+1_ and S_i+2_, when γ_i_ = 0°, and (**d**) Top view of the arrangement of two adjacent loops, L_i_ and L_i+1_, and three adjacent β-strands, S_i_, S_i+1_ and S_i+2_, when γ_i_ = ±180°.

In this section, we compare both the β-γ plots for β-barrels^TM^ and β-barrels^cytoplasm^ at the macroscopic level in analogy with Ramachandran ϕ-ψ plot at the atomistic level to find out the distribution density of dihedral angles. Our β-γ plot reflects the Ramachandran map of peptide distribution, such as favorable and unfavorable regions for β-strands and loops as given in Fig. 3(a,b). The overall distribution can possibly assist to understand the similarities and dissimilarities in their structural arrangement patterns. Overall β and γ distributions are restricted to certain regions for β-barrels^TM^ indicating they show conformationally similar arrangements that are observed in Ω-λ plot for TM helical proteins^37^. On the other hand, β and γ distributions for β-barrels^cytoplasm^ are sparsely distributed over greater regions signifying dissimilar patterns in their arrangements than the membrane counterparts. Conventionally, the relative conformation of adjacent structural elements i.e. neighboring elements both at residues and secondary structures level have influence on each other and that can be identified by the distribution of their dihedral angles^44–46^. Our new joint-derived dihedral angles such as β and γ distributions were related to the structural arrangement of β-stands for both β-barrels^TM^ and β-barrels^cytoplasm^ architecture just as previously demonstrated with TM helical proteins using Ω and λ dihedral angles^37^. The dihedral angle between the joints is not only associated with the arrangements of the individual β-strands and loops but also involves in the dependency of adjacent secondary structural element orientations. If β-strands and loops are simplified as shown in Fig. 1, β-strands arrangements can be explained by β type dihedral angles between the i^th^ β-strand (S_i_) and its neighboring i + 1^th^ β-strand (S_i+1_). Similarly, the type γ can represent the arrangement of the loop segments between the i^th^ loop (L_i_) and its neighboring i + 1^th^ loop (L_i+1_). In addition, type γ dihedral angle also provides the relative arrangement between the i^th^ β-strand (S_i_) and i + 2^th^ β-strand (S_i+2_). Figure 4 show some specific relation between the dihedral angles and β-strands (or loops) arrangements. When the dihedral angle β_i_ is close to 0 degrees, β-strand S_i_ is antiparallel with the adjacent β-strand S_i+1_ (Fig. 4(a)). In a similar way, when the dihedral angle β_i_ is close to ±180 degrees, β-strand S_i_ is parallel with the adjacent β-strand S_i+1_ (Fig. 4(b)). When the dihedral angle γ_i_ is close to 0 degrees, loop L_i_ is antiparallel with the adjacent loop L_i+1_, and β-strand S_i_ and β-strand S_i+2_ are on the same side with respect to β-strand S_i+1_ (Fig. 4(c)). Meanwhile, when the dihedral angle γ_i_ is close to ±180 degrees, loop L_i_ is parallel with the adjacent loop L_i+1_, and β-strand S_i_ and β-strand S_i+2_ are on the opposite side respect to β-strand S_i+1_ (Fig. 4(d)). Here, we attempt to compare both β-barrels^TM^ and β-barrels^cytoplasm^ geometric characteristics through consecutive β-strands and loops arrangements by investigating the distribution of β and γ dihedral angles.

**Figure 5:**
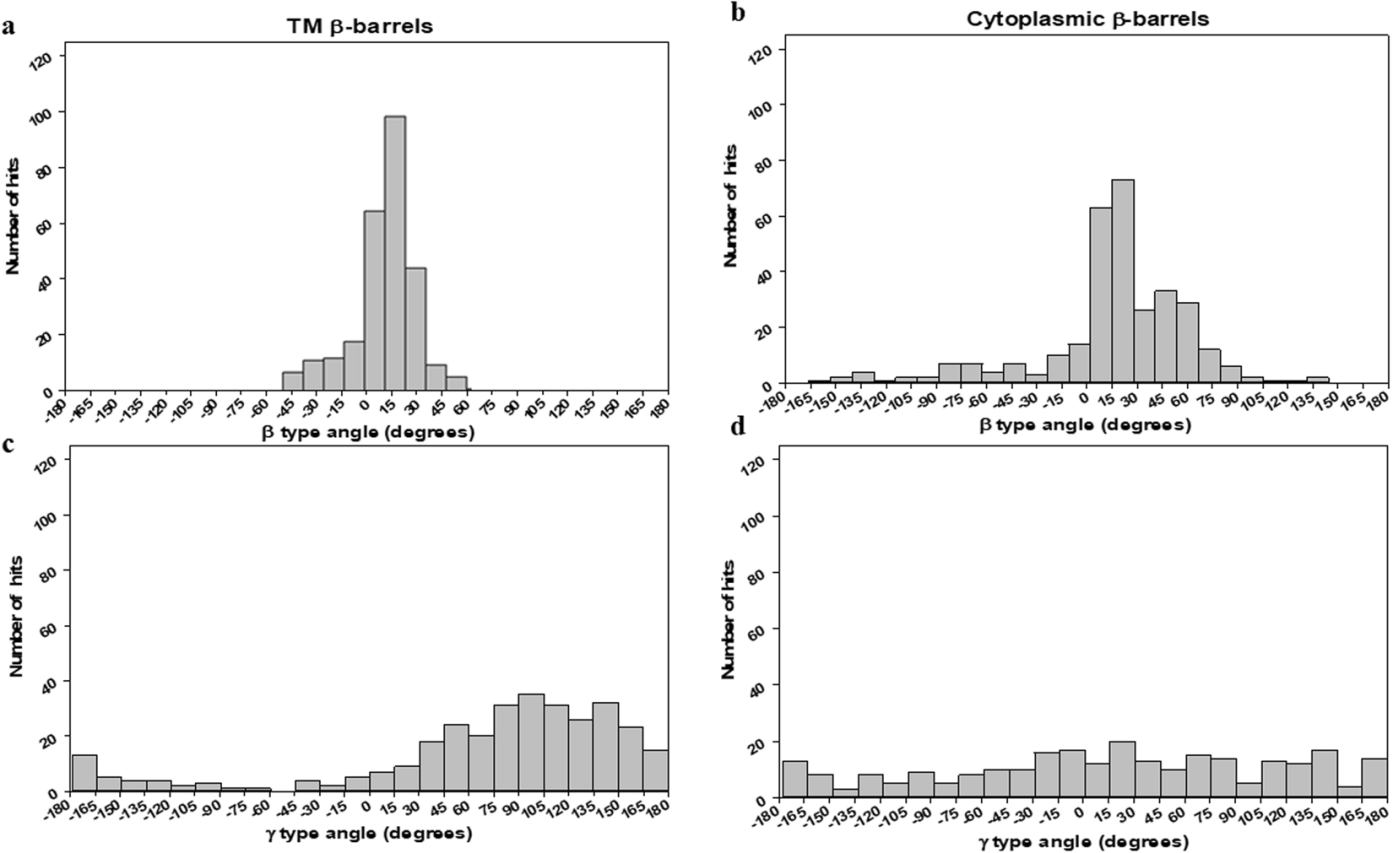
The overall distribution of β and γ dihedral angles. (**a**) Histogram showing the distribution of β dihedral angles for β-barrels^TM^ proteins. (**b**) Histogram showing the distribution of β dihedral angles for β-barrels^cytoplasm^ proteins. (**c**) Histogram showing the distribution of γ dihedral angles for β-barrels^TM^ proteins. (**d**) Histogram showing the distribution of γ dihedral angles for β-barrels^cytoplasm^ proteins.

The overall distribution plots for all β and γ angles that belong to both β-barrels^TM^ (29) and β-barrels^cytoplasm^ (51) non-homologous protein structures was shown in Fig. 5. In both cases, it was evident that the β type dihedral angles were mostly confined to the range of −30° to +30°. Figure 5(a) shows more than 80% of β distribution between 0° to +30° for β-barrels^TM^, where the frequency in a clockwise (positive angles) orientation is evidently dominant than that of anti-clockwise (negative angles). We observed the most favored dihedral angle distribution regions between +10° and +20° corresponding to their restricted distribution space for TM β dihedral angles. Meanwhile, β type distributions for β-barrels^cytoplasm^ were observed in an almost entire range of −150° to +150° with a strong preference towards 0° to +30° as given in Fig. 5(b). In contrast, type γ dihedral angles were distributed in the entire possible region between *−*180° to +180° for both β-barrels^TM^ and β-barrels^cytoplasm^ proteins. However, TM γ distribution shows dominant peaks between +90° to +180° region (Fig. 5(c)). Possibly, γ preference of TM structures observed to be very dominance between +100° to +150° considering the overall distribution region. While type γ dihedral angles for β-barrels^cytoplasm^ are distributed widely with equal preference (Fig. 5(d)). Interestingly, the frequencies of TM γ type dihedral angles in clockwise orientations are apparent than counter-clockwise, suggesting a preferable arrangement of adjacent β-strands tend to be right-handed orientation. Together, TM β-strands topological arrangements differ from cytoplasmic β-strands in overall β-barrel architecture, i.e. both β and γ dihedral angle distributions vary with one another. Membrane β-strands incline to pack within smaller conformational space, whereas cytoplasmic β-strand arrangements were relatively different. The propensities for antiparallel and right-handed orientation properties of β-strands for TM and soluble proteins are reported in several proteins packing studies^47,48^. Certainly, both β-barrels^TM^ and β-barrels^cytoplasm^ share related conformational distribution space with significant variations observed in the cytoplasmic structures.

In terms of arrangements, Fig. 5(a,b) show β type dihedral angles indicated the preference in the narrow range as a main accessible region that two neighboring β-strand (S_i_ and S_i+1_) most likely to be arranged in antiparallel as depicted in Fig. 4. It should also be noted that cytoplasmic β-strand arrangements show antiparallel, parallel and mixed arrangements in the β-barrel proteins. Despite the sparse distribution in the all possible range of −180° to +180°, it is observed that TM γ-type dihedral angles were predominantly distributed between 30° to +180° region according to Fig. 4(c). However, the overall orientations of L_i_ and L_i +1_ indicate that β-barrels^TM^ neighboring loops are appeared to exist in both anti-parallel and parallel arrangements. On the other hand, cytoplasmic γ-type dihedral angles do not have a strong preference towards any region supporting the all possible antiparallel, parallel and mixed arrangements. Thus, cytoplasmic β-strand S_i+2_ are freely arranged between the same side and opposite side to β-strand S_i_.

### Comparison of local patterns

A comparison of local patterns of TM β-barrels with cytoplasmic β-barrel geometries was attempted by analyzing the nearest neighbor frequencies of β and γ-type dihedral angles. The measurement of dihedral angle β_i_ enables the prediction of the arrangement of neighboring β-strands (S_i_ and S_i+1_) and the relative positions of two β-strands S_i_ and S_i+2_ can be determined by measuring dihedral angle γ_i_. These indicate that the measurements of the continuous dihedral angles can allow us to predict how the β-strands in β-barrel proteins are continuously arranged and to which extent they are twisted and varied. For instance, the information of β_i_ and β_i+1_ can determine the arrangement of S_i_, S_i+1_, and S_i+2_, and the information of γ_i_ and γ_i+1_ may allow the prediction of the relative positions of S_i+2_ and S_i+3_ to S_i_ and S_i+1_. Here, to study the β-strands arrangements in both β-barrels^TM^ and β-barrels^cytoplasm^ systems, we examined the local patterns of continuous dihedral angle clusters such as β_i_ − β_i+1_, γ_i_ − γ_i+1_, β_i_ − β_i+1_ − β_i+2_ and γ_i_ − γ_i+1_ − γ_i+2_. Most of the β-strand structures in β-barrels^TM^ were observed with (+, +) and (+, +, +) patterns for β_i_ − β_i+1_, γ_i_ − γ_i+1_, and the same trend have been traced with signature patterns of β-barrels^cytoplasm^ as well. Interestingly, (+, +) signature pattern means a strong bias toward right-handed topology.

**Figure 6:**
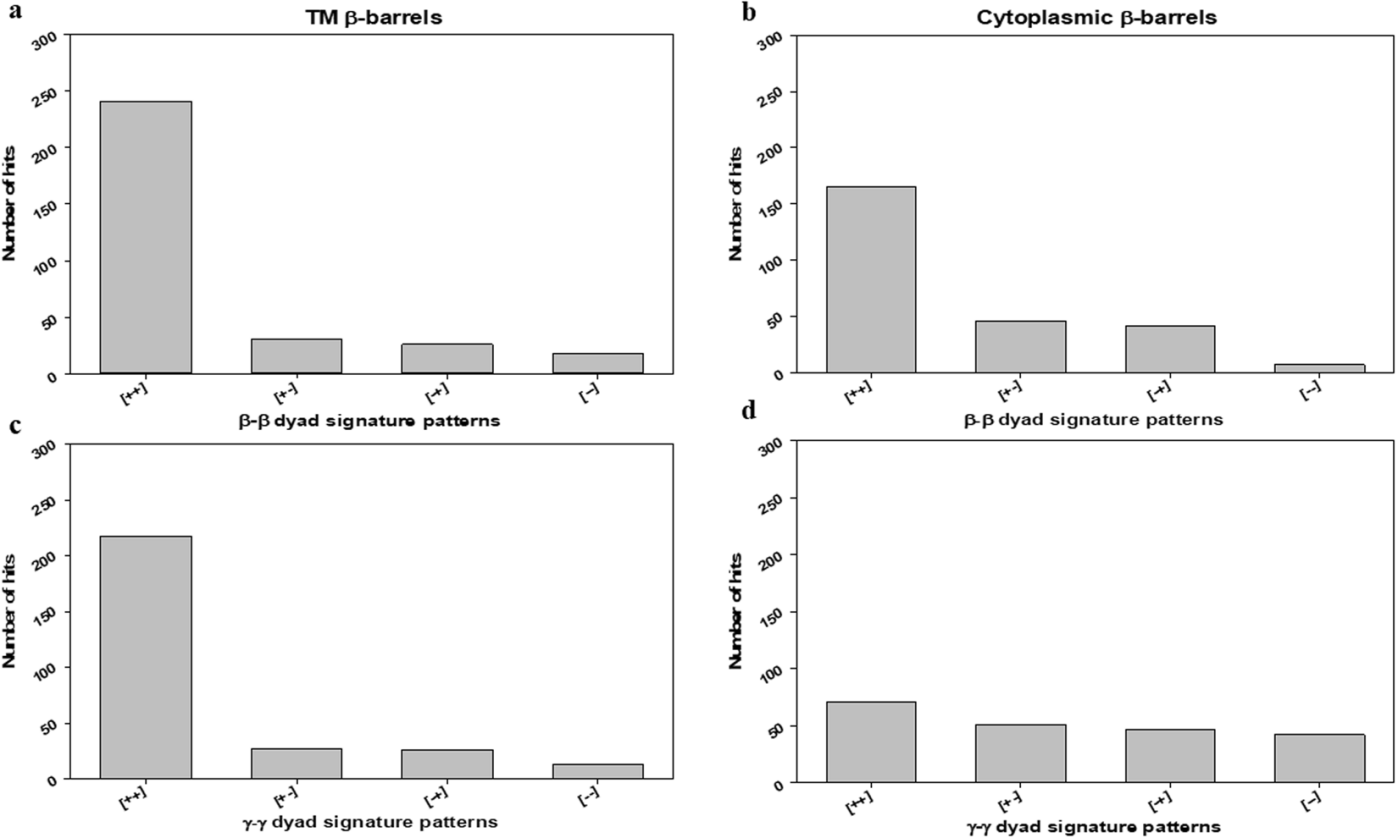
Frequencies of the dyad patterns for consecutive β or γ type dihedral angles when β or γ type angles are categorized as (+) and (−). The bar diagrams show the observed numbers of (**a**) four different patterns of two consecutive β type angles, β_i_ − β_i+1_ for β-barrels^TM^ proteins. (**b**) β-barrels^cytoplasm^ proteins showing four different patterns of two consecutive β type angles, β_i_ − β_i+1_ (**c**) Four different patterns of two consecutive γ type angles, γ_i_ − γ_i+1_, for β-barrels^TM^ proteins. (**d**) β-barrels^cytoplasm^ proteins showing four different patterns of two consecutive γ type angles, γ_i_ − γ_i+1_.

**Figure 7:**
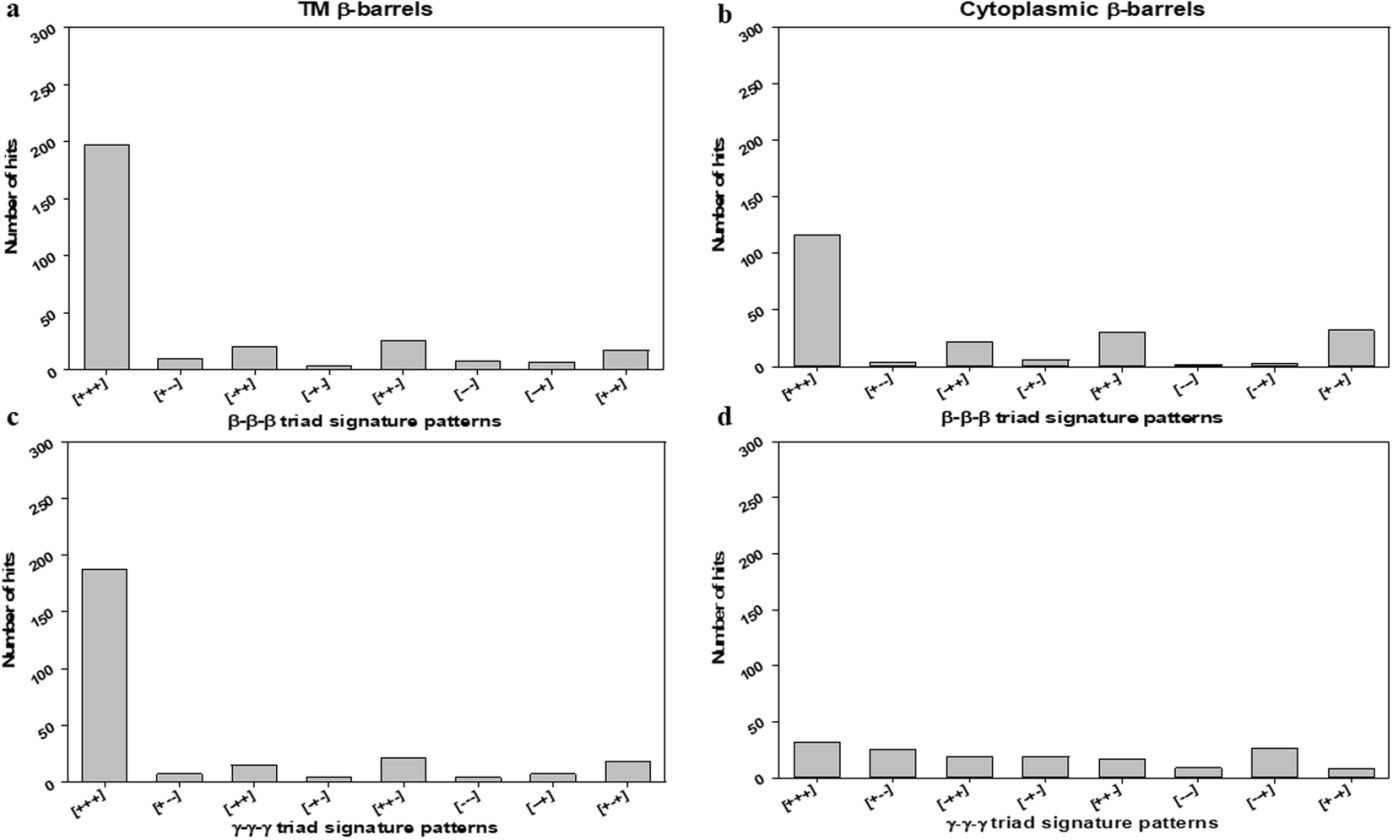
Frequencies of the triad patterns for consecutive β or γ type dihedral angles when β or γ type angles are categorized as (+) and (−). The bar diagrams show the observed numbers of (**a**) eight different patterns of three consecutive β type angles, β_i_ − β_i+1_ − β_i+2_, for β-barrels^TM^ proteins. (**b**) β-barrels^cytoplasm^ proteins eight different patterns of three consecutive β type angles, β_i_ − β_i+1_ − β_i+2_. (**c**) Eight different patterns of three consecutive γ type angles, γ_i_ − γ_i+1_ − γ_i+2_, for β-barrels^TM^ proteins. (**d**) β-barrels^cytoplasm^ proteins eight different patterns of three consecutive γ type angles, γ_i_ − γ_i+1_ − γ _i+2_.

**Figure 8:**
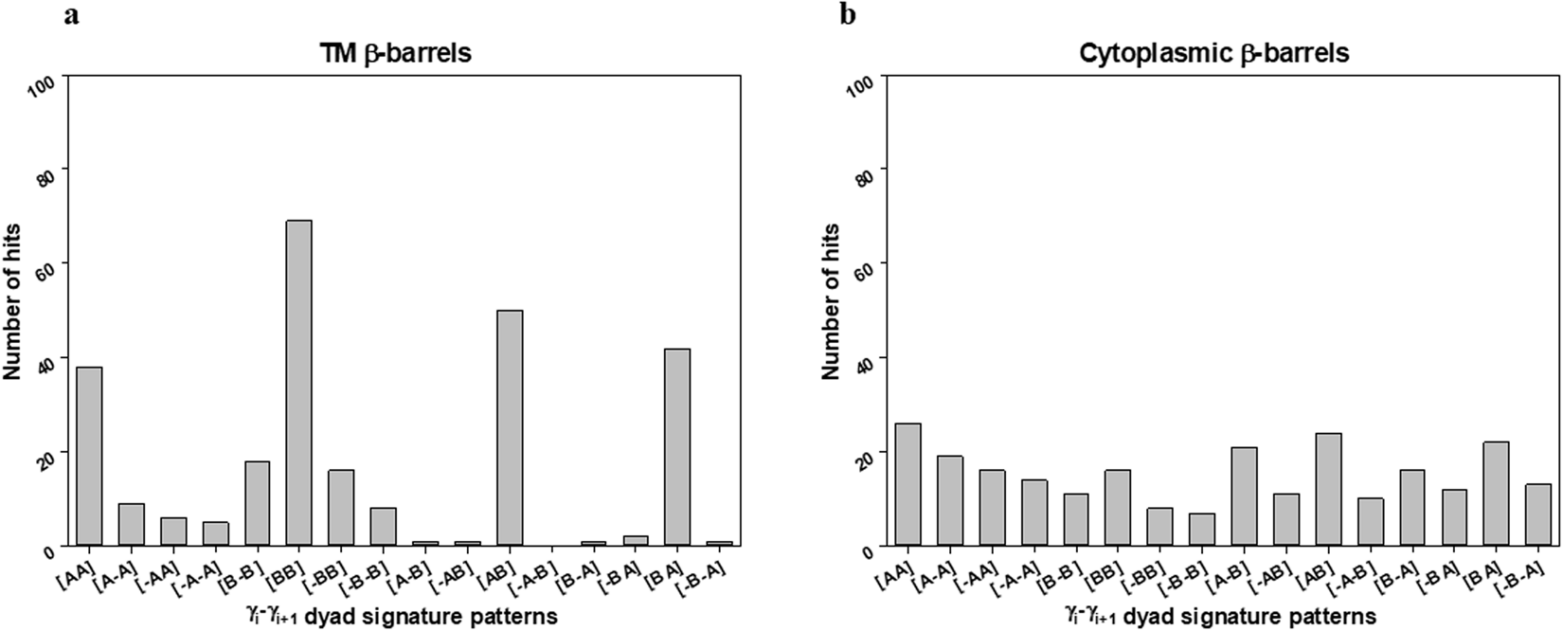
Frequencies of the dyad patterns for consecutive γ type dihedral angles when the γ type dihedral angle was split into four regions. The bar diagram shows the observed numbers of the 16 different patterns of two consecutive γ type angles, γ_i_ − γ_i+1_. Here, all λ type angles were split into four regions, i.e. ^+^A(0° to 90°), ^+^B(90° to 180°), ^−^A(− 90° to 0°), and ^−^B (−90° to −180°) (**a**) Out of 16 patterns from the combinations of two consecutive γ type angles, γ_i_ − γ_i+1_, [BB] pattern were abundant for β-barrels^TM^ proteins (29) and (**b**) for β-barrels^cytoplasm^ proteins, there is no obvious preference in the patterns in their 51 non-homologous dataset.

Dihedral angles of β and γ-type were categorized into two groups: clockwise - positive value (from 0 to +180 degrees) and counter-clockwise - negative value (from −180 to 0 degrees). Then, the dihedral angles (β and γ) for both β-barrels^TM^ (29) and β-barrels^cytoplasm^ (51) structures were interpreted as the combination of signatures (positive and negative sign) (SI Tables (5, 6, 7 and 8)). Firstly, dyad signature (i.e. Two adjacent/consecutive units) distribution patterns of β_i_ − β_i+1_ and γ_i_ − γ_i+1_ for both datasets was examined and that indicated a strong tendency towards (+, +) pattern (Fig. 6). For a β_i_ − β_i+1_ cluster, the frequency of (+, +) pattern was obviously higher than others for both β-barrels^TM^ and β-barrels^cytoplasm^, respectively (Fig. 6(a,b)). Also, the frequency of (+, +) pattern was higher than others for the γ_i_ − γ_i+1_ cluster (Fig. 6(c)). Only cytoplasmic γ_i_ − γ_i+1_ has shown a moderately different trend in all their groups (Fig. 6(d)). For example, the pattern (*−*, *−*) showing considerable frequency, whereas all other dyad clusters have it as the least common. Secondly, we examined the triad sequential signature patterns (i.e. three adjacent/consecutive units) of β type and γ type dihedral angles for both β-barrels^TM^ and β-barrels^cytoplasm^ structures. We observed β_i_ − β_i+1_ − β_i+2_ and γ_i_ − γ_i+1_ − γ_i+2_ clusters predominant towards (+, +, +) patterns among the 8 possible combinations (Fig. 7(a–c)). And patterns such as (*−*, +, +), (+, +, *−*), and (+, *−*, +) were also observed noticeably. However, cytoplasmic γ_i_ − γ_i+1_ − γ_i+2_ (Fig. 7(d)) has been identified with relatively different inclination in all their signature patterns. Finally, the comparisons of γ-type dihedral angles of TM with cytoplasmic proteins were performed. Both γ-type distributions were found in the entire range from *−*180° to +180°. Subsequently, the dihedral angle space was divided into four quadrants as (I) ^+^A: 0° to +90°, (II) ^−^A: *−*90° to 0°, (III) ^+^B: +90° to +180°, and IV) ^−^B: *−*180° to *−*90° for further adjacent frequency distribution analysis. In the quadrant, the pattern of γ_i_ − γ_i+1_ cluster was studied more in detail. Among all possible 16 combinations of γ_i_ − γ_i+1_ cluster, the most abundant dihedral angle distribution was observed in the range of (+90° to +180°, +90° to +180°) for β-barrels^TM^ (Fig. 8(a)). There is no such clear preference for β-barrels^cytoplasm^. However, there are few positive (AA), (BB), (AB) and (BA) regions with relatively better propensities were observed commonly in both the cases. The observed β-strands and loop angles are intensely positive, corresponding to a right-handed twist orientation. Altogether, type β_i_ − β_i+1_ and γ_i_ − γ_i+1_ signature analysis has revealed that the positive sign as a clearly dominant region for adjacent β-strands which can be correlated to the right-handed twist.

**Figure 9:**
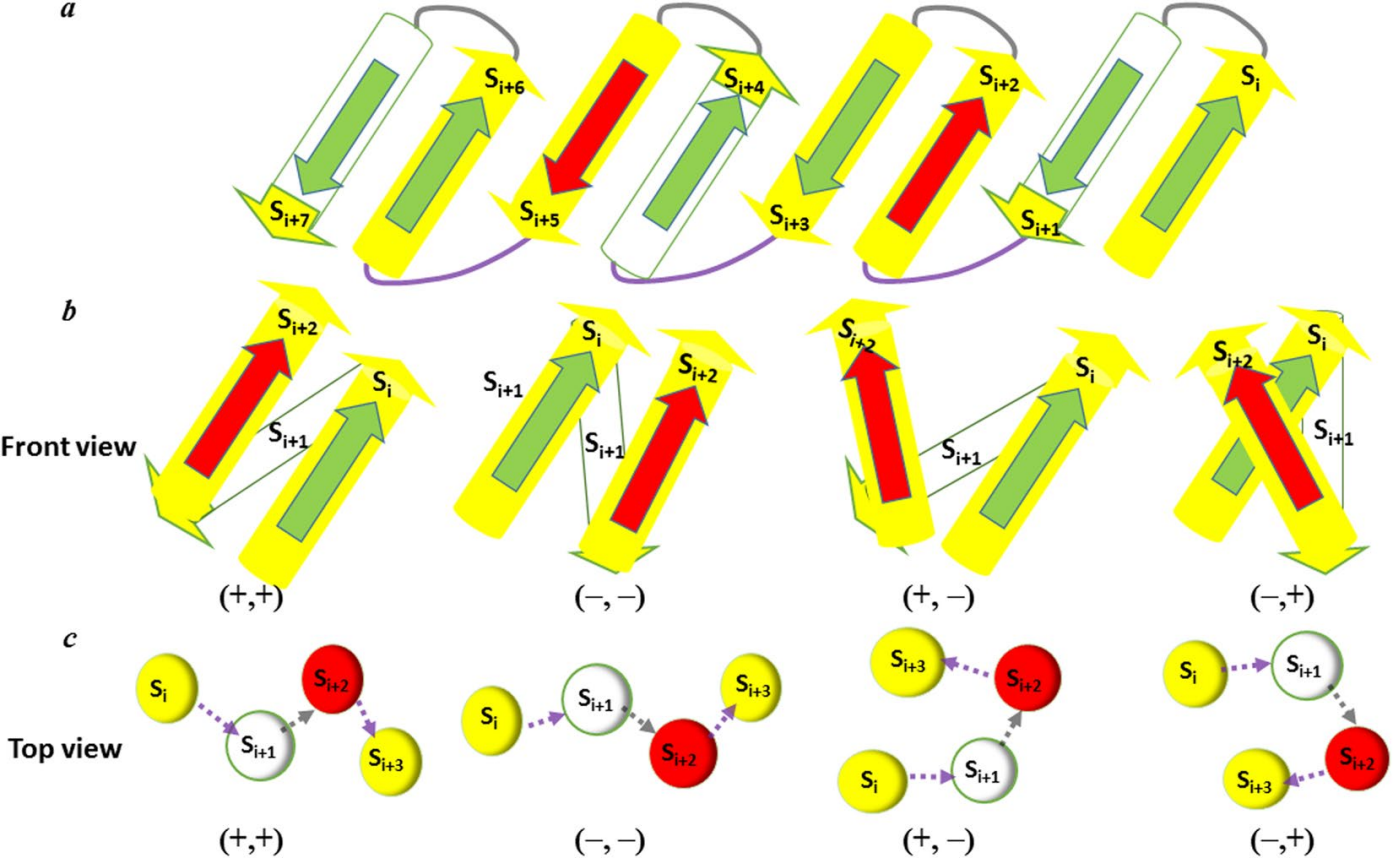
Relation to the dihedral angle patterns and β-strands arrangements or extensions. (**a**) Front view of the linearly ordered β-strands in β-barrel proteins with β-strands and loops. (**b**) Side view of the arrangement of three consecutive β-strands S_i_ − S_i+1_ − S_i+2_ depending on the four different patterns of two consecutive β type angles, β_i_ − β_i+1_. (**c**) Top view configuration of four consecutive helices, S_i_ − S_i+1_ − S_i+2_ − S_i+3_, for the four different patterns of two consecutive γ type angles, γ_i_ − γ_i+1_.

The twisted property of β-strands can be explained by both the conformation of individual β-strands and the relative orientations of adjacent β-strands using β and γ type dihedral angles. Figure 9 shows a conventional image of β-barrel proteins with several β-strands that have previously used to portray adjacent TM helical arrangements. Figure 9(a,b) show the schematic arrangement of three consecutive β-strands, i.e. S_i_, S_i+1_, and S_i+2_, depending on the pattern of β_i_ − β_i+1_ cluster, observed in the front and side view respectively. As recorded in Fig. 4, four different (+, +), (+, −), (−, +) and (−, −) patterns determine four different types of arrangement between S_i_ and S_i+2_ in parallel (Fig. 9(b)). The dominance of (+, +) and (+, +, +) patterns in β_i_ − β_i+1_ and β_i_ − β_i+1_ − β_i+2_ cluster indicates that β-barrel favor twisted pattern. Figure 9(c) shows a schematic picture of β-barrel proteins with several β-strands in top view. It shows how the β-strands were extended depending on the pattern of γ_i_ − γ_i+1_ cluster. The (−, −) and (+, +) patterns suggest that β-strands are set in one direction with a distorted pattern in such a way it forms a twisted topology. The (+, *−*) and (−, +) patterns, however, show that β-strands are arranged such that β-strands are tightly packed. The clear preference of (+, +) and (+, +, +) pattern for γ_i_ − γ_i+1_ and γ_i_ − γ_i+1_ − γ_i+2_ clusters suggests that β-strands in the membrane proteins favor the arrangements with a twisted pattern in the right-hand direction. Similarly, the dominant dihedral angular distribution of (90° to 180°, 90° to 180°) in the 16 possible patterns of γ_i_ − γ_i+1_ cluster (Fig. 8) implies that there is also some angle preference in the twisted type arrangement of TM β-strands than cytoplasmic ones. These dihedral angle preferences and the right-handed properties of the β-strands are strongly interrelated with the overall β-barrel architectures. The specific conformations of β_i_ − β_i+1_ and γ_i_ − γ_i+1_ cluster confirms that adjacent positive angles may provide continued stability, thus β-strands twisting possibly control the conformational space.

The right-handed orientation and antiparallel arrangement of β-barrels^TM^ can be related to the distribution of β type and γ-type dihedral angles found between +10° to +20° and +100° to +150° respectively. Few TM γ-type dihedral angles show a trend of relatively small dihedral value for short intracellular loops and larger dihedral value for long extracellular loops in their distributions locally, whereas no such bias has observed with β-barrels^cytoplasm^ proteins. In general, conformational energetics strongly favors the antiparallel β-strand arrangements which have an advantage over the stability in terms of the packing interaction^49^. It is also known that the antiparallel barrel folds are particularly more stable when the strand orders are a consecutively right-handed conformation^50^. The combined orientations of individual strands and adjacent strands adopt an overall closed barrel or partially opened or distorted barrels. Structurally, the right-handed connectivity is common for all β-strands embedded in the finite hydrophobic membranes that prefer the antiparallel arrangements. Meanwhile, no specific trends have been found with β-barrels^cytoplasm^ proteins in terms of their γ-type. Both TM and cytoplasmic adjacent loop orientations were observed as they can be found opposite side with no direct interaction between them. Though loops are arranged in a random orientation that can lead to maximization of entropy, they adopt few recurrent conformations (adjacent loops orientations can be specific for protein families). Besides, we subsequently quantified the respective dihedral angle distributions in effect with the number and position of β-strands for both β-barrels^TM^ and β-barrels^cytoplasm^ proteins.

### Comparison of β-strand numbers and positions effect in both β-barrels

**Figure 10:**
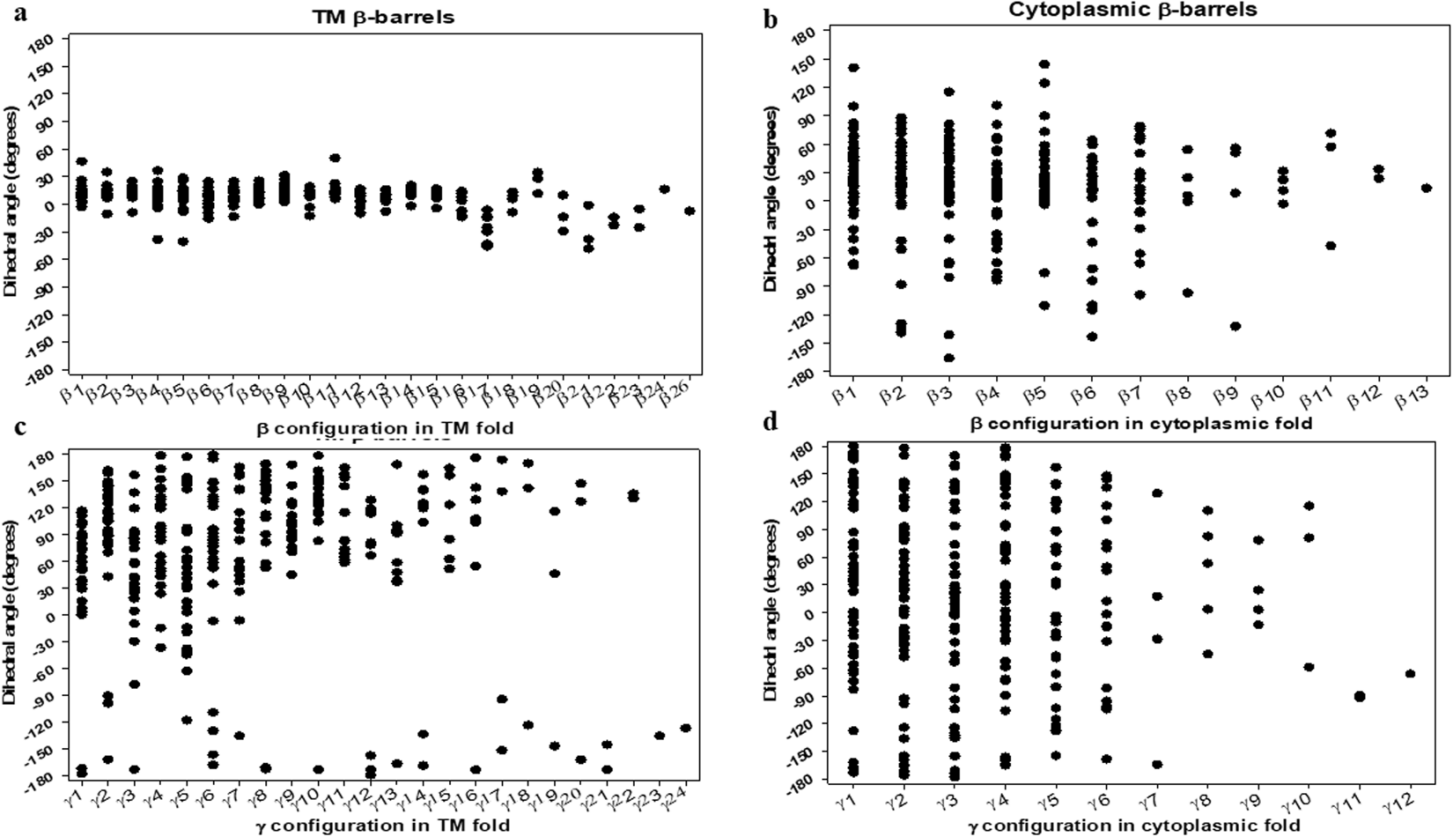
Dihedral angle distributions in the β-barrels^TM^ and β-barrels^cytoplasm^ proteins depending on their configurations. (**a**) Scatter plot of the β type dihedral angles of β-barrels^TM^ proteins depending on their configurations. (**b**) Scatter plot of β type dihedral angles of β-barrels^cytoplasm^ proteins depending on their configurations. (**c**) Scatter plot of the γ type dihedral angles of β-barrels^TM^ proteins depending on their configurations. (**d**) Scatter plot of γ type dihedral angles of β-barrels^cytoplasm^ proteins depending on their configurations. For (**a**–**d**), all the i^th^ dihedral angles (β_i_ or γ_i_ values) of both of β-barrels^TM^ (29) and β-barrels^cytoplasm^ (51) non-homologous β-barrel proteins were collected and plotted against β_i_ or γ_i_ in the x-axis.

**Figure 11:**
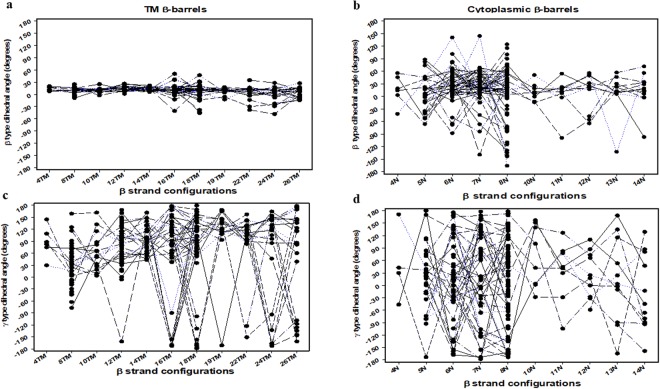
Dihedral angle distributions in the β-barrels^TM^ and β-barrels^cytoplasm^ proteins depending on the β-strands numbers. (**a**) Scatter and line plot of the β type dihedral angles of β-barrels^TM^ proteins depending on the β-strands numbers. (**b**) Scatter and line plot of β type dihedral angles of β-barrels^cytoplasm^ proteins depending on the β-strands numbers. (**c**) Scatter and line plot of the γ type dihedral angles of β-barrels^TM^ proteins depending on the β-strands numbers. (**d**) Scatter and line plot of γ type dihedral angles of β-barrels^cytoplasm^ proteins depending on the β-strands numbers. For (**a**–**d**), all the i^th^ dihedral angles (β_i_ or γ_i_ values) of both β-barrels^TM^ (29) and β-barrels^cytoplasm^ (51) non-homologous β-barrel proteins were given in y-axis and plotted against β-strands numbers in the x-axis.

We also examined how the similarities and dissimilarities for both β-barrels^TM^ and β-barrels^cytoplasm^ in terms of the number and position of β-strands. As the numbers of β-strands vary, the arrangements of β-strands and loops might be changed due to change of interaction energy between β-strands and entropy of the flexible loop region. The distributions of dihedral angles for β and γ types were plotted according to their relative positions in β-strands for TM fold (Fig. 10(a,c)) and cytoplasmic fold (Fig. 10(b,d)) respectively. It seems β-barrels^TM^ proteins are relatively larger than β-barrels^cytoplasm^ proteins, and so the numbers of β-strands are observed less in cytoplasmic folds. According to the number of β-strands, the β and γ type dihedral distribution were plotted for TM configurations (Fig. 11(a,c)) and cytoplasmic configurations (Fig. 11(b,d)) respectively. For β-barrels^TM^ proteins with 4, 10, 12, 14 and 19 β-strands obviously suggest that γ type distribution is also restricted to specific accessible regions and on the other hand, rather sparse distribution noted with 16, 18, 22 and 26 β-strands (Fig. 11(c)). For β-barrels^cytoplasm^ proteins with 5, 6, 7 and 8 β-strands were found to be the major groups determining their distributions (Fig. 11(b)). In both cases, the distribution of β and γ type dihedral angles was quite different compared to those of overall distributions as shown in Fig. 5. These results suggest that the dihedral angles are significantly affected by the relative position of β-strands and loops as the number of the β-strands fluctuate in both groups. Further, the patterns of the distribution of β_i_ − β_i+1_ and γ_i_ − γ_i+1_ cluster were analyzed according to three different numbers of groups for both cases.

**Figure 12:**
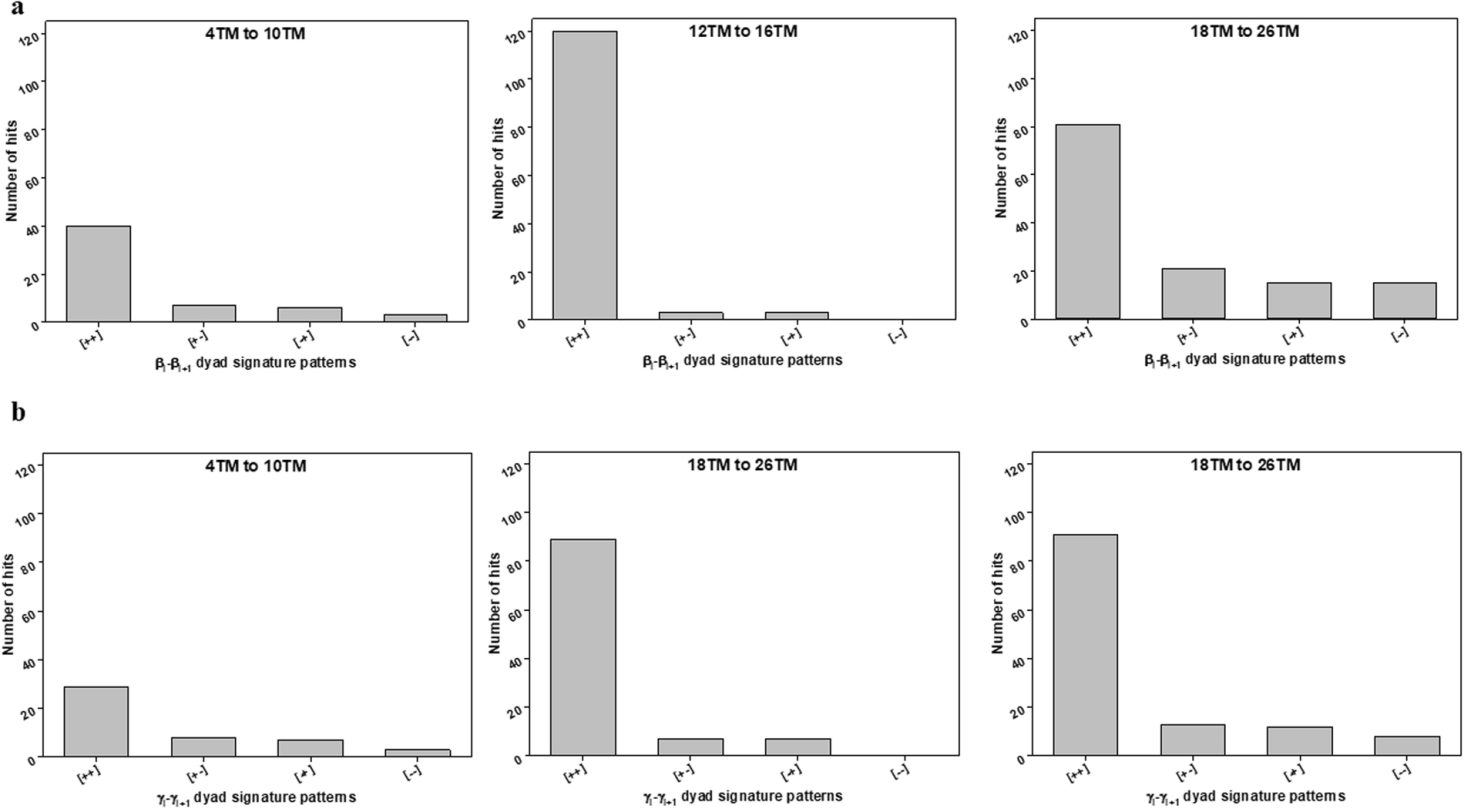
Frequencies of the patterns for two consecutive β or γ type dihedral angles depending on the β-barrels^TM^ proteins with a different number of β-strands. All the 29 non-homologous β-barrels^TM^ proteins were categorized into three groups, i.e., proteins with 4 to 10 TM β-strands, 12 to 16 TM β-strands, and 18 to 26 TM β-strands. The bar diagrams show the observed numbers of (**a**) four different patterns of two consecutive β type angles, β_i_ − β_i+1_, in each group, and (**b**) four different patterns of two consecutive γ type angles, γ_i_ − γ_i+1_, in each group.

**Figure 13:**
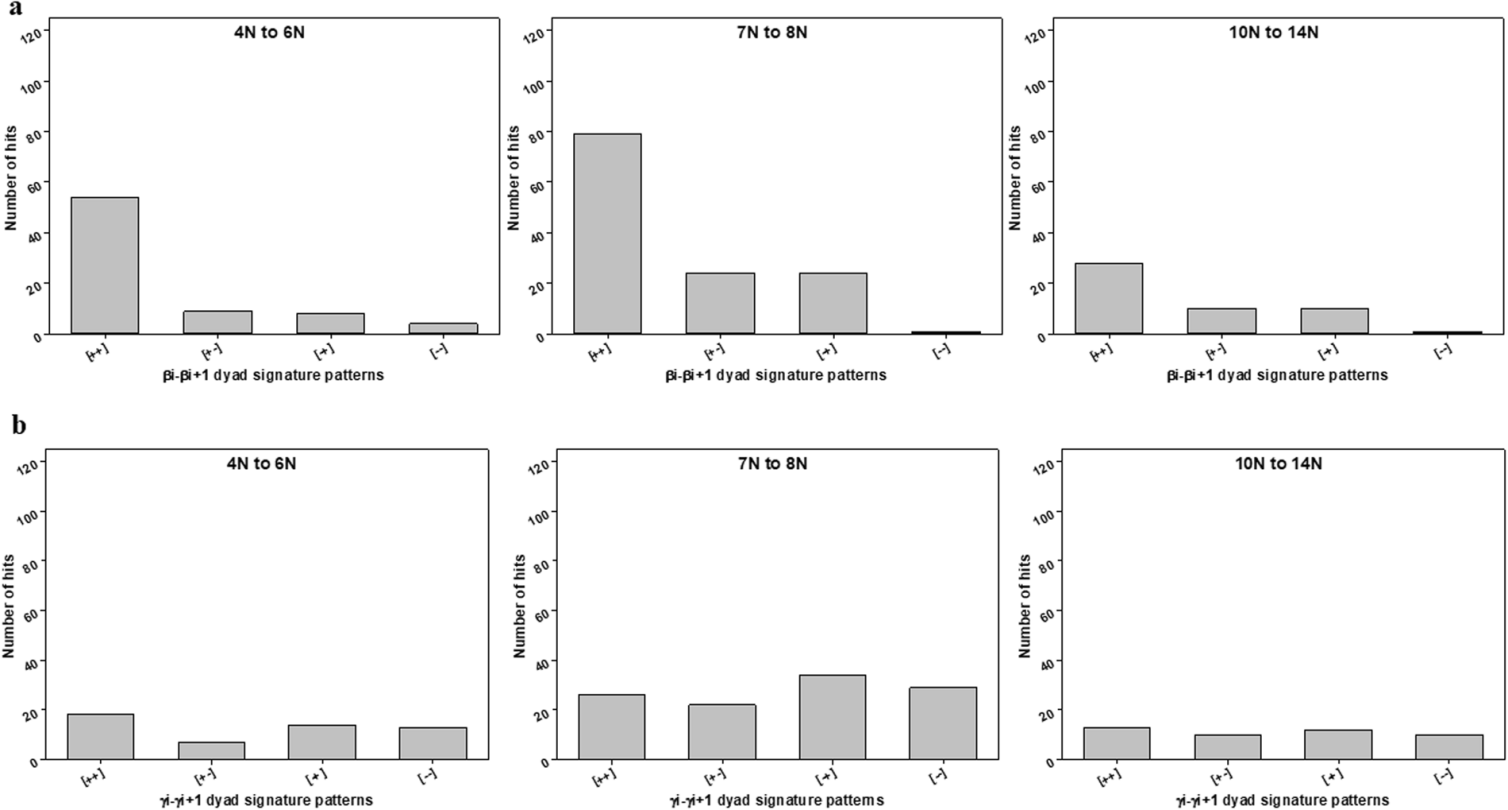
Frequencies of the patterns for two consecutive β or γ type dihedral angles depending on the β-barrels^cytoplasm^ proteins with a different number of β-strands. All the 51 non-homologous β-barrels^cytoplasm^ proteins were categorized into three groups, i.e., proteins with 4 to 6N β-strands, 7 to 8N β-strands, and 10 to 14N β-strands. The bar diagrams show the observed numbers of (**a**) four different patterns of two consecutive β type angles, β_i_ − β_i+1_, in each group, and (**b**) four different patterns of two consecutive γ type angles, γ_i_ − γ_i+1_, in each group.

Membrane β-barrel structures with 4–10 TM β-strands, with 12–16 TM β-strands, and with 18–26 TM β-strands were considered exclusively for size effect and grouped into smaller (structures with 4–10 TM β-strands), medium (structures with 12–16 TM β-strands) and larger membrane (structures with 18–26 TM β-strands) β-barrel proteins (Fig. 12(a,b)). In contrast, cytoplasmic β-barrel structures were grouped into 4–6N β-strands, 7–8N β-strands, and 10–14N β-strands according to their size grouping, where N denotes the number of β-strands (Fig. 13(a,d)). As shown in Fig. 12(a), (+, +) pattern for β_i_ − β_i+1_ cluster show up more frequently for the proteins with 4–10 TM β-strands. The pattern remained same as the TM increases from 4–10 TM β-strands to 12–16 TM β-strands and for 18–26 TM β-strands. However, it is found that (*−*, +), (+, *−*) and (−, −) patterns also shows up notably with 18–26 TM β-strands group compared to other groups. Dihedral angles of β_17_, β_20_ and β_21_ are sparsely distributed in the negative angles (−60° to 0°) belongs to β-barrel proteins with 18–24 TM β-strands. The (+, +) pattern becomes more dominant with all group proteins sharing β-strands. Especially, pattern (+, +) shows up more frequently for the proteins with 4–10 TM β-strands for the γ_i_ − γ_i+1_ cluster. As the number of TM β-strands increase, it becomes more prevail in 12–16 TM β-strands and 18–26 TM β-strands groups (Fig. 12(b)). Noticeably, the (+, +) pattern is abundant for all the TM β-strands groups. Though (+, +) pattern is dominant, (*−*, +), (+, *−*) and (*−*, *−*) patterns are also observed considerably for β_i_ − β_i+1_ and γ_i_ − γ_i+1_ clusters of the proteins with larger TM structures (18–26 TM β-strands), respectively. On the other hand, cytoplasmic β-barrel proteins show slightly different trends. Although cytoplasmic β_i_ − β_i+1_ also follows the same (+, +) pattern as the preferred pattern in all respective groups as observed with TM proteins (Fig. 13(a)) and the patterns trend for γ_i_ − γ_i+1_ cluster are different. As shown in Fig. 13(b), for γ_i_ − γ_i+1_ cluster, (+, *−*) pattern is the least common pattern of the small proteins with 4–6N β-strands. However, the pattern shifts as the β-strands (N) increases from 4–6N β-strands to 7–8N β-strands, where (+, *−*) pattern become dominant, yet other patterns such as (+, +), (*−*, +) and (*−*, *−*) are also observed substantially. For 10–14N β-strands, all (+, +), (+, *−*), (*−*, +) and (*−*, *−*) patterns remains equally dominant. Despite the overall uniformity in their structural arrangements, these results imply that β-strands arrangement of both β-barrel structures prefers to be positioned in a specific pattern for the efficient packing of β-strands as the number of β-strands changes. Like the overall distribution, the dihedral angles (β and γ) and signature preferences for various β-strands numbers and position are rather different between β-barrel proteins in the membrane and cytoplasmic origin.

## Discussion

The present study provides a systematic comparison of macroscopic features of β-barrels^TM^ and β-barrels^cytoplasm^ proteins, providing a more simplistic way to understand the structural organization of complex proteins. Furthermore, our studies help to differentiate the specificity through various β-strands arrangements by analyzing the overall conformational space, signature patterns, positions and numbers of β-strands. For the comparative analysis of TM β-barrels with cytoplasmic structures, the joint-derived dihedral angles of β-strands were used. The β-γ dihedral angles are macroscopic descriptions of protein structures based on joint-based approach^37,38^. In both cases, specific allowed and disallowed conformational accessible spaces were found. Here, we used non-homologous datasets of β-barrels^TM^ and β-barrels^cytoplasm^ proteins for comparison and the approach can be extended to compare the conformational heterogeneities and geometric features of other polymers. It is well known that the adjacent β-strands in the membrane were mostly arranged in antiparallel due to a confinement inside membrane favoring stability and conformational energetics^49,50^. The overall dihedral angle distribution analyses for the β and γ-type suggest that the neighboring two β-strands would be antiparallel orientation as shown in Fig. 3(a) for TM proteins. On the other hand, cytoplasmic β-strands with greater conformational flexibility can possibly have all the antiparallel, parallel and mixed arrangements. In addition, the analyses of adjacent dihedral signature pattern analysis, such as β_i_ − β_i+1_ and γ_i_ − γ_i+1_ intensely supports the TM β-strands favored to be right-handed orientations with (+, +) pattern being clearly dominant. For cytoplasmic proteins, the same (+, +) signature patterns are clearly abundant preferring the right-handed orientations. Commonly, the (+, +) patterns were dominant over other signatures for β-strands arrangements. Though γ-type overall distribution is different in both the cases, they have common features of (+, +) patterns for loop-loop arrangements. Based on the results, we envisage that right-handed orientation is profoundly relevant to the stereochemistry of interaction between β-strands at the atomic level. Presumably, the successive combination of (+, +) pattern for β_i_ − β_i+1_ (orientations of β-strands) and γ_i_ − γ_i+1_ (orientations of loops) has an advantage for TM β-strands packing. With relatively greater conformational space, cytoplasmic proteins tend to have slightly loosely packed β-strands. Together, these results prove the packing similarities and dissimilarities between β-barrels^TM^ and β-barrels^cytoplasm^ proteins.

The difference in the overall dihedral angle distributions according to the number of β-strands for both the β and γ-type reveals additional specifics. We support the fact that the β-strands positioning of β-barrel proteins could be significantly restricted more inside membrane lipid bilayer than the cytoplasmic environment as the number of TM β-strands changes. Our results could aid to comprehend the differences in their overall structure as it is reflected in the variability of the dihedral angle distributions of β and γ-type at the macroscopic level. This infers the local arrangement of β-strands and loops can be affected and varied by the number of β-strands increases. The γ_i_ − γ_i+1_ cluster analysis also shows a clear preference of a “right-handed” conformation as the number of β-strands fluctuates, implying a right-handed orientation is optimized form required for the efficient TM β-strand packing geometry. In contrast, (+, *−*) pattern appear marginally higher than (+, +) pattern of the cytoplasmic proteins with 4–6N β-strands. It was clearly observed that the arrangements of β-strand orientations are diverse depending on the β-strand positions in cytoplasmic proteins. Taken together, our results indicate that the β-barrel structure in the lipid membrane environment is more significantly controlled than the structures of cytoplasmic β-strand structures at local and global arrangements.

All such macroscopic geometric analyses provide significant clues for better understanding to improve in-silico protein structure and function prediction, modeling, and validation. The relationship between the dihedral angles and β-strand arrangements provide more focus on β-barrels engineering and designing principles. In general, any rule that is related to such structural property supports to compare and classify the protein functions. Here, the antiparallel arrangements and right-handed orientations are common for most of the β-strand structures, while the mixed loop arrangement is also essential for β-barrel structural formation. Perhaps, non-adjacent and various combinations of β and γ-type dihedral angles would be distinguished to locate interaction and non-interaction part of the β-strands. In future, the combination of macroscopic joint description of protein structures as a coarse-grain level with all-atom or Cα residue level can help us to improve our understanding towards large structural rearrangements and dynamic behavioral studies of complex structures.

## Methods

### Datasets as target β-barrel proteins used in this study

At first, we selected and filtered all membrane proteins from the Protein Data Bank by using the key word “Membrane proteins” and then, with the help of selection mode, β-barrel proteins X-ray crystal structures were separated. It was found that 837 structures belong to TM β-barrel proteins. The dataset of 592 β-barrel structures having sequence identity less than 90%, were identified to be unique proteins containing both homologous and non-homologous protein chains. From the above numbers, the search was made for a non-homologous dataset with the following criteria (1) sequence identity less than 30%, (2) ≤3.0 Å resolution structures, (3) selection of monomeric protein chains were chosen manually from homomeric structures when more than a single chain is available, (4) removal of remote homologous i.e. when more than one structure is available for the superfamily, to avoid the structural redundancy we removed all structures that share same superfamily and kept single structure to represent the superfamily. Finally, a total of 29 proteins satisfying all the above criteria were grouped as non-homologous membrane proteins. The above selection processes were done with the help of PDB and PISCES server^51,52^. Obtained TM β-barrel proteins with TM β-strands from 4TM to 26TM were classified using PPM server with OPM^53^ and PDBTM^54^ databases. Then, the Protein Data Bank database was used to acquire a list of all structures available of cytoplasmic β-barrels. From all beta proteins, only closed or partly opened β-barrel structures were chosen using CATH AND SCOP annotations^55–57^. To make certain the same analyses, structures with X-ray resolution lower than 3.0 Å with sequence identity less than 30% were considered with the help of PISCES server. From the list of 117 structures of β-barrel proteins, we removed the remote homologous and we obtained 51 structures as a final cytoplasmic dataset. We extracted the secondary structure such as β-strands and loops assignments from the obtained structures using STRIDE server^58^. The same were cross-referenced with the SHEET records in the PDB header information for cytoplasmic β-barrel proteins.

### Joint-based structural joint points and β and γ-type dihedral measurements

The first and last residue Cα atoms of each TM and cytoplasmic secondary structural (SS) segment were projected as joint structural points for the dihedral calculations. Structural joint selections and β-strands boundaries were prepared based on the annotations given in OPM and STRIDE server respectively. The previously developed in-house program was used for the joint-derived dihedral angle measurements. It should be noted that the total number of β and γ-type dihedral angles for a protein structure entirely depends on the total number of TM β-strands and loops present in the structure i.e. the total number of SS segments and joint coordinates. While forming all linkage of the joint points, a macroscopic description of the overall protein structure was portrayed.

### Statistical analyses dihedral angle patterns

To find out the preferred relative orientations, the conformational analysis was performed based on the dihedral angles obtained and the signature patterns dominance was revealed among various combinations of consecutive dihedral angles. For the selected 29 and 51 structures for both TM and cytoplasmic cases presented by the β_n_-γ_n_-β_n+1_-γ_n+1_ dihedral angle set, as summarized in Supplementary Information Tables (S1, S2, S3 and S4); N stands for β strand numbers in the protein structures. The calculated dihedral angles were converted to positive (+ve) and negative (−ve) signatures to represent the conformations, as given in Supplementary Information Tables (S5, S6, S7 and S8). A consecutive β-β pattern was selected for each fold as β_n_-β_n+1_. Grouped β_n_-β_n+1_ should be a consecutive, adjacent set, and no fixed order, whereas non-consecutive β_n_-β_n+2_ were not considered. For example, the β-β pattern angles were selected from β_1_-γ_1_- β_2_-γ_2_- β_3_-γ_3_ to β_n_-γ_n_ as any consecutive β_n_-β_n_. To make more defined distribution patterns, the consecutive β_n_-β_n+1_ and β_n_-β_n+1_-β_n+2_ were also tested.

## Electronic supplementary material

Supplementary Information
