# Supplementary material for: Comparative Analysis of TM and Cytoplasmic β-barrel Conformations Using Joint Descriptor
Source: Sci Rep. 2018 Sep 21;8:14185. doi: 10.1038/s41598-018-32136-4 (PMC6155101; doi:10.1038/s41598-018-32136-4)
Supplement: Supplementary file 1 — Supplementary Information [file 41598_2018_32136_MOESM1_ESM.pdf]

Supplementary Information

Comparative Analysis of TM and Cytoplasmic β-barrel Conformations Using Joint Descriptor

Jayaraman Thangappan[a], Sangwook Wu[a, *] and, Sun-Gu Lee[b, *]

[a]Department of Physics, Pukyong National University, Busan, 608-737, Republic of Korea

[b]Department of Chemical Engineering, Pusan National University, Busan, 609-735, Republic of Korea

Corresponding Authors:

Sangwook Wu

Department of Physics, Pukyong National University, Busan, 608-737, Republic of Korea

Telephone: +82-51-629-5578

E-mail: sangwoow@pknu.ac.kr

Sun-Gu Lee

Department of Chemical Engineering, Pusan National University, Busan, 609-735, Republic of Korea

Telephone: +82-51-510-2786

E-mail: sungulee@pusan.ac.kr

Table S1: Measured β type dihedral angles for all 29 β-barrels^{TM} proteins.

SI#	TM#	PDB	β1	β2	β3	β4	β5	β6	β7	β8	β9	β10	β11	β12	β13	β14	β15	β16	β17	β18	β19	β20	β21	β22	β23	β24	β25
1	4TM	1EK9A	19.8	16.97	18.02																						
2	4TM	2GR8A	7.81	11.59	7.27																						
3	4TM	3X2RA	10.27	17.38	9.23																						
4	8TM	1P4TA	15.67	12.22	13.99	6.91	-7.86	4.6	7.46																		
5	8TM	1QJPA	8.33	10.23	7.53	-0.69	3.8	4.11	3.87																		
6	8TM	2ERVB	10.44	11.51	-8.95	-1.71	-5.83	11.43	12.41																		
7	8TM	2X27X	6.73	14	15.46	-1.3	5.08	9.14	4.84																		
8	8TM	3DZMA	3.92	10.85	9.25	-3.75	-5.1	7.87	-2.28																		
9	8TM	3GP6A	16.93	14.64	13.23	3.29	-5.68	24.77	19.14																		
10	10TM	2VDFA	11.88	11.8	14.04	11.72	10.35	-2.28	7.71	10.95	10.93																
11	10TM	2X55A	10.03	13.91	25.16	3.53	5.91	10.4	13.79	8.43	11.32																
12	12TM	1QD6C	19.83	8.99	15.05	11.91	15.99	8.7	24.99	25.53	20.75	19.49	7.37														
13	12TM	1TLYA	11.61	16.17	8.06	11.49	17.53	5.78	15.01	10.1	18.68	12.04	10.99														
14	12TM	1UYNX	8.2	20.81	9.37	11.78	14.09	8.77	7.93	13.18	5.87	12.21	8.08														
15	12TM	2WJRA	26.19	10.28	17.35	7.41	9.4	10.81	14.52	16.66	18.67	9.18	12.11														
16	12TM	3FIDA	13.07	8.06	12.53	12.41	26.22	1.34	6.4	13.26	12.21	8.14	13.13														
17	12TM	4RL8A	11.01	9.22	8.28	24.67	9.55	13.82	16.24	12.44	20.04	14.68	17.74														
18	14TM	2X9KA	15.98	10.06	15.68	7.14	13.44	5.94	18.83	21.47	18.59	12.05	11.21	11.35	9.66												
19	14TM	3BS0A	9.09	7.71	10.61	17.82	9	18.57	9.24	15.46	10.68	15.59	11.17	11.81	10.76												
20	16TM	2FGQX	1.95	35.13	19.37	36.58	-40.76	10.58	16.15	3.09	17.33	-12.51	50.38	8.76	16.01	12.69	12.83										
21	16TM	4C00A	9.99	8.66	10.81	7.71	17.65	8.86	16.35	20.01	2.63	-3.08	10.37	11.64	7.08	20.54	16.99										
22	16TM	4Y25A	3.44	14.57	13.15	14.69	10.95	0.15	18.05	11.32	10.39	14.58	12.23	16.78	5.66	9.1	14.68										
23	18TM	1A0TP	11.68	16.94	20.02	16.61	14.77	-4.18	14.03	10.3	13.83	10.43	6.02	3.46	6.94	10.23	9.01	3.88	-13.73								
24	18TM	2YNKA	46.78	-10.52	19.26	-38.31	14.55	4.11	9.22	-0.09	31.6	-3.24	14.87	9.32	2.9	14.1	11.96	11.94	-43.33								
25	18TM	3SZVA	10.58	13.68	18.66	6.73	28.88	-15.51	6.84	10.85	23.14	9.3	22.66	-9.81	15.11	11.03	13.96	-13.61	-45.89								
26	19TM	4C69X	10.54	10.36	10.12	13.73	17.49	7.4	11.98	4.46	6.79	15.01	10.59	13.9	5.31	13.06	9.36	-12.5	-5.99	5.85							
27	22TM	1FEPA	-2.74	6.92	12.46	6.63	13.8	14.45	2.31	16.63	7.39	9.58	11.71	-3.37	15.74	12.87	6.64	14.34	-29.82	9.15	34.7	-13.75	-38.14				
28	24TM	3FIPA	6.79	7.04	10.57	18.08	12.28	9.63	4.25	8.45	13.92	10.99	14.37	2.38	-7.78	-1.86	-4.29	-6.79	-24.75	13.32	28.04	-29.08	-48.37	-22.59	-25.22		
29	26TM	4Q35A	15.32	10.86	16.44	15.38	-6.62	-9.31	-13.31	6.75	27.26	11.8	12.52	9.91	10.11	17.46	8.03	7.87	-14.48	-8.61	11.68	10.02	-1.15	-13.89	-5.17	16.44	-7.34

Table S2: Measured γ type dihedral angles for all 29 β-barrels^{TM} proteins.

SI#	TM#	PDB	γ1	γ2	γ3	γ4	γ5	γ6	γ7	γ8	γ9	γ10	γ11	γ12	γ13	γ14	γ15	γ16	γ17	γ18	γ19	γ20	γ21	γ22	γ23	γ24
1	4TM	1EK9A	74.64	109.03																						
2	4TM	2GR8A	29.5	82.48																						
3	4TM	3X2RA	88.16	144.7																						
4	8TM	1P4TA	72.63	116.47	25.02	43.24	-44.26	91.11																		
5	8TM	1QJPA	15.8	70.05	-29.43	51.65	-19.05	58.87																		
6	8TM	2ERVB	3.34	43.21	-77.46	-14.37	-42.47	84.62																		
7	8TM	2X27X	38.06	159.02	30.9	49.42	-13.58	62.73																		
8	8TM	3DZMA	34.33	125.26	4.62	24.1	-37.56	52.82																		
9	8TM	3GP6A	7.79	78.48	-9.4	-36.37	-62.58	-6.64																		
10	10TM	2VDFA	64.75	115.29	18.55	66.36	9.01	87.57	26.57	81.44																
11	10TM	2X55A	50.58	161.96	27.72	33.14	3.45	132.34	50.48	53.24																
12	12TM	1QD6C	0.34	88.96	57.81	83.41	63.86	126.98	50.36	161.53	100.7	83.03														
13	12TM	1TLYA	114.7	-161.62	74.86	99.39	33.62	80.09	44.55	142.26	126.6	113.19														
14	12TM	1UYNX	39.85	134.89	59.01	97.64	15.17	34.99	-5.86	57.85	45.2	115.84														
15	12TM	2WJRA	101.71	149.55	40.93	88.11	41.17	96.25	53.29	160.29	70.654	104.71														
16	12TM	3FIDA	87.11	113.37	94.12	140.77	29.95	70.7	37.64	108.93	92.13	129.17														
17	12TM	4RL8A	79.85	91.81	19.24	123.72	97.38	129.37	103.39	168.54	100.79	147.46														
18	14TM	2X9KA	86.14	130.8	57.56	100.01	46.43	77.33	60.51	90.35	94.06	161.84	64.81	128.86												
19	14TM	3BS0A	52.77	90.77	42.92	119.29	60.15	148.2	95.25	140.19	86.02	148.09	73.7	114.17												
20	16TM	2FGQX	37.78	-89.98	-172.66	178.48	177.16	-156.03	156.00	150.43	111.04	-172.79	83.72	81.17	58.98	125.11										
21	16TM	4C00A	104.27	131.85	119.46	132.28	53.78	83.62	114.98	-170.26	168.11	178.51	61.82	66.86	37.33	103.83										
22	16TM	4Y25A	91.43	116.74	85.28	151.92	151.76	175.03	139.94	-172.14	111.65	115.35	58.85	78.47	48	139.56										
23	18TM	1A0TP	102.94	148.44	81.17	122.35	149.83	148.92	83.89	136.71	88.09	126.87	157.52	-178.96	91.86	120.91	51.72	54.82								
24	18TM	2YNKA	-177.26	-98.66	156.8	163.6	72.69	179.6	157.72	169.28	101.26	154.15	68.92	117.03	100.73	124.83	62.69	103.76								
25	18TM	3SZVA	101.06	139.99	35.4	59.16	140.92	-129.42	141.2	156.47	75.95	114.76	164.97	-157.03	92.86	-168.28	156.16	-172.97								
26	19TM	4C69X	-171.37	115.78	137.1	93.34	146.32	121.48	165.73	112.86	145.11	134.31	144.21	113.83	168.52	119.64	164.65	107.74	138.32							
27	22TM	1FEPA	111.49	95.31	90.45	130	92.94	141.15	104.4	129	105.13	122.76	82.89	119.07	94.88	157.49	85.08	142.74	-151.09	-122.99	116.05	127.01				
28	24TM	3FIPA	116.93	105.14	85.34	142.55	154.21	-167.75	95.95	146.13	123.1	150.97	153.95	-172.72	-166.13	-133.12	164.55	129.21	-94.06	142.02	46.39	147.05	-172.91	130.65		
29	26TM	4Q35A	60.12	81.34	86.14	178.52	-117.5	-108.69	-134.91	145.72	84.62	142.09	114.81	116.41	38.88	140.62	123.55	176.01	173.76	169.86	-146.51	-161.71	-144.84	136.14	-134.93	-126.45

Table S3: Measured β type dihedral angles for all 51 β-barrels^{cytoplasm} proteins.

SI#	TM#	PDB	β1	β2	β3	β4	β5	β6	β7	β8	β9	β10	β11	β12	β13
1	4N	2B97A	45.64	-42.01	18.92										
2	4N	1G3PA	55.53	14.34	2.91										
3	5N	1NB9A	-52.9	-2.3	-2.04	-13.05									
4	5N	1WHIA	42.81	-51.29	0	7.26									
5	5N	1PV4A	41.95	18.56	46.84	3.62									
6	5N	1GCPA	46.06	16.99	16.55	-41.22									
7	5N	1HK9A	22.41	41.28	19.89	-14.94									
8	5N	1R6JA	-66.23	19.63	73.92	-10.9									
9	5N	1O6AA	82.43	88.05	3.81	3.1									
10	5N	1G31A	20.11	5.22	-39.99	-35.01									
11	6N	2JDID	20.54	62.2	42.47	6.28	58.64								
12	6N	1WJXA	140.63	25	45.03	80.67	-1.69								
13	6N	1AGJA	24.23	33.63	65.81	29.09	6.5								
14	6N	1DFUP	-68.14	16.42	43.86	101.09	2.21								
15	6N	1S98A	3.72	-88.24	14.62	67.21	5.16								
16	6N	1EFTA	48.73	35.61	14.36	20.58	2.31								
17	6N	2D9RA	15.33	61.22	20.56	10.84	7.67								
18	6N	2IMLA	-9.98	51.35	81.22	5.23	3.1								
19	6N	1BCOA	46.83	16.15	58.52	20.07	4.04								
20	6N	1TS9A	55.5	22.34	16.15	-50.68	5.22								
21	6N	2BLNA	28.36	82.44	28.9	11.04	43.06								
22	6N	2F1LA	51.06	16.9	15.07	-65.14	8.25								
23	7N	1WPOA	68.57	17.31	17.55	65.88	22.81	-84.2							
24	7N	1IK9A	18.2	23.78	16.01	39.42	17.29	9.95							
25	7N	1ORUA	43.44	-139.3	67.28	38.27	14.86	59.25							
26	7N	1JEYA	18.72	60.48	-66.78	51.84	27.83	10.36							
27	7N	1RQPA	24.98	47.95	25.75	-15.25	17.49	64.36							
28	7N	1O70A	32.16	61.62	30.03	20.62	43.41	3.41							
29	7N	1J0WA	22.62	36.29	18.58	-44.53	8.81	13.39							
30	7N	2GUJA	8.03	5.14	60.48	19.77	144.28	17.75							
31	7N	1QZ8A	-14.84	29.49	53.51	17.89	-3.74	9.9							
32	8N	1EAR	56.08	9.91	54.51	19.27	124.32	27.93	-65.99						
33	8N	1UE0A	16.18	71.55	41.72	15.53	-110.64	-143.41	64.02						

34	*8N*	*1A1XA*	*25.29*	*40.41*	*15.33*	*-80.29*	*23.73*	*41.9*	*12.15*					
35	*8N*	*1NYCA*	*36.5*	*18.5*	*-80.77*	*18.87*	*22.87*	*9.76*	*30.55*					
36	*8N*	*1OEWA*	*55.24*	*11.41*	*53.4*	*-75.68*	*73.23*	*-22.76*	*65.43*					
37	*8N*	*1XE1A*	*4.24*	*57.87*	*44.23*	*53.96*	*26.33*	*-43.87*	*68.26*					
38	*8N*	*1T2WA*	*15.82*	*-129.41*	*34.35*	*53.75*	*22.97*	*-71.7*	*0.24*					
39	*8N*	*1PQHA*	*76.77*	*56.88*	*20.63*	*30.93*	*89.91*	*-22.4*	*50.29*					
40	*8N*	*2CPLA*	*-1.89*	*76.45*	*17.16*	*-83.84*	*8.83*	*-115.07*	*76.12*					
41	*8N*	*1Y12A*	*100.08*	*50.94*	*115.16*	*64.8*	*6.55*	*12.46*	*-10.93*					
42	*8N*	*2Q03A*	*-40.26*	*17.72*	*8.31*	*6.67*	*0.43*	*11.76*	*8.51*					
43	*8N*	*2F9HA*	*54*	*-134.17*	*50.52*	*33.07*	*52.64*	*-110*	*78.73*					
44	*8N*	*1BEBA*	*61.7*	*17.72*	*3.1*	*33.63*	*35.74*	*11.43*	*13.89*					
45	*8N*	*1GQBA*	*23.82*	*-5.08*	*-165.97*	*38.29*	*19.18*	*41.45*	*23.98*					
46	*8N*	*1F3UB*	*-30.26*	*60.82*	*-141.35*	*34.27*	*-75.93*	*25.92*	*-29.12*					
47	*10N*	*2P12A*	*18.99*	*25.68*	*-14.46*	*6.87*	*6.58*	*10.75*	*-12.27*	*-0.57*	*50.96*				
48	*11N*	*2FR2A*	*17.74*	*-2.73*	*7.52*	*22.33*	*21.16*	*12.22*	*-99.35*	*54.29*	*8.25*	*11.19*			
49	*12N*	*4FGFA*	*51.18*	*23.15*	*-64.63*	*17.06*	*49.43*	*21.95*	*-55.77*	*24.68*	*55.58*	*31.56*	*-47.42*		
50	*13N*	*1NLSA*	*8.76*	*7.49*	*30.32*	*12.45*	*10.42*	*46.11*	*12.54*	*5.76*	*-132.47*	*22.46*	*56.98*	*23.92*	
51	*14N*	*1H4GA*	*19.11*	*-2.49*	*6.51*	*12.06*	*17.21*	*34.37*	*28.83*	*-97.12*	*55.85*	*-2.98*	*71.49*	*33.64*	*13.52*

Table S4: Measured γ type dihedral angles for all 51 β-barrels^{cytoplasm} proteins.

SI#	TM#	PDB	γ1	γ2	γ3	γ4	γ5	γ6	γ7	γ8	γ9	γ10	γ11	γ12
1	*4N*	*2B97A*	*-46.39*	*29.79*										
2	*4N*	*1G3PA*	*170.87*	*42.62*										
3	*5N*	*1NB9A*	*179.93*	*-92.81*	*110.84*									
4	*5N*	*1WHIA*	*39.72*	*50.52*	*0*									
5	*5N*	*1PV4A*	*-173.04*	*34.8*	*-15.41*									
6	*5N*	*1GCPA*	*36.61*	*83.93*	*4.81*									
7	*5N*	*1HK9A*	*30.6*	*113.3*	*22.56*									
8	*5N*	*1R6JA*	*70.57*	*169.79*	*29.37*									
9	*5N*	*1O6AA*	*-74.02*	*16.59*	*-81.14*									
10	*5N*	*1G31A*	*75.2*	*-33.73*	*-19.28*									
11	*6N*	*2JDID*	*-167.53*	*-159.08*	*19.84*	*167.98*								
12	*6N*	*1WJX*	*-24.97*	*134.67*	*-0.94*	*-30.71*								
13	*6N*	*1AGJ*	*33.87*	*-154.7*	*-130.8*	*-13.79*								
14	*6N*	*1DFUP*	*-11.2*	*35.61*	*-104.04*	*-28.06*								
15	*6N*	*1S98A*	*-82.97*	*-16.65*	*50.96*	*16.28*								
16	*6N*	*1EFTA*	*165.53*	*-40.61*	*140.76*	*143.97*								
17	*6N*	*2D9RA*	*144.51*	*-164.86*	*15.79*	*175.8*								
18	*6N*	*2IMLA*	*-36.73*	*-136.15*	*-135.46*	*30.74*								
19	*6N*	*1BCOA*	*141.12*	*-35.37*	*9.62*	*115.65*								
20	*6N*	*1TS9A*	*22.56*	*93.3*	*26.54*	*169.95*								
21	*6N*	*2BLNA*	*0.95*	*-33.8*	*134.22*	*-164.85*								
22	*6N*	*2F1LA*	*45.24*	*124.91*	*73.72*	*68.59*								
23	*7N*	*1WPOA*	*-172.97*	*-25.52*	*160.11*	*-4.49*	*-115.09*							
24	*7N*	*1IK9A*	*75.71*	*114.11*	*21.91*	*20.73*	*88.68*							
25	*7N*	*1ORUA*	*-4.68*	*77.93*	*-124.06*	*71.93*	*-154.76*							
26	*7N*	*1JEYA*	*-42.51*	*15.38*	*-32.04*	*-155.93*	*-3.6*							
27	*7N*	*1PJ7A*	*140.45*	*83.84*	*-3.2*	*145.53*	*121.6*							
28	*7N*	*1RQRA*	*168.13*	*-175.78*	*135.15*	*-106.01*	*-66.39*							
29	*7N*	*1J0WA*	*129.19*	*139.32*	*21.15*	*64.7*	*139.73*							
30	*7N*	*2GUJA*	*60.59*	*-124.19*	*157.83*	*12.21*	*-49.07*							
31	*7N*	*1QZ8A*	*-65.63*	*177.74*	*-177.82*	*-7.52*	*-121.63*							
32	*8N*	*1EARA*	*-161.8*	*57.97*	*-124.61*	*178.25*	*-21.62*	*-14.31*						
33	*8N*	*1UE0A*	*52.01*	*-98.77*	*-53.45*	*-58.78*	*70.94*	*69.46*						
34	*8N*	*1A1X*	*116.98*	*-2.39*	*-135.51*	*56.19*	*111.26*	*-15.5*						
35	*8N*	*1NYCA*	*-55.82*	*121.41*	*93.64*	*95.33*	*87.46*	*135.3*						
36	*8N*	*1OEWA*	*23.04*	*43.46*	*131.42*	*-52.43*	*-46.54*	*12.31*						
37	*8N*	*1XE1A*	*173.19*	*-171.5*	*-170.32*	*2.02*	*-26.12*	*-104.09*						
38	*8N*	*1T2WA*	*119.95*	*90.35*	*-132.89*	*-159.1*	*118.86*	*-101.5*						
39	*8N*	*1PQHA*	*-127.76*	*-165.93*	*10.01*	*-164.59*	*-103.25*	*143.98*						
40	*8N*	*2CPLA*	*-62.5*	*4.46*	*-173.27*	*-73.47*	*-127.91*	*49.6*						
41	*8N*	*1Y12A*	*-19.68*	*-21.19*	*119.11*	*168.83*	*-22.9*	*-1.67*						
42	*8N*	*2Q03A*	*141.91*	*141.48*	*61.81*	*-20.07*	*137.63*	*115.47*						
43	*8N*	*2F9HA*	*-10.47*	*79.91*	*169.86*	*27.52*	*33.78*	*74.68*						

44	***8N***	***1BEBA***	***60.96***	***25.72***	***-45.15***	***149.18***	***65.35***	***147.9***						
45	***8N***	***1GQBA***	***112.86***	***-47.91***	***-52.39***	***92.89***	***120.36***	***-81.55***						
46	***8N***	***1F3UB***	***136.39***	***65.14***	***-155.14***	***-71.69***	***49.8***	***45.39***						
47	***10N***	***2P12A***	***151.55***	***1.78***	***42.27***	***139.62***	***156.95***	***100.35***	***-28.42***	***3.63***				
48	***11N***	***2FR2A***	***44.46***	***77.73***	***40.93***	***126.72***	***29.69***	***-104.41***	***-28.85***	***82.53***	***78.23***			
49	***12N***	***4FGFA***	***87***	***-27.95***	***-0.13***	***73.19***	***66.19***	***-31.17***	***17.51***	***110.3***	***24.43***	***-59.09***		
50	***13N***	***1NLSA***	***168.41***	***24.05***	***-2.3***	***134.7***	***-10.34***	***-95.51***	***-164.37***	***53.25***	***3.18***	***115.27***	***-89.55***	
51	***14N***	***1H4GA***	***47.31***	***86.7***	***-93.92***	***-89.42***	***-80.34***	***-158.42***	***129.02***	***-44.88***	***-13.08***	***80.91***	***-91.87***	***-66.16***

Table S5: Converted signs of β type dihedral angles for the 29 β-barrels^{TM} proteins.

SI#	*TM#*	*PDB*	*β1*	*β2*	*β3*	*β4*	*β5*	*β6*	*β7*	*β8*	*β9*	*β10*	*β11*	*β12*	*β13*	*β14*	*β15*	*β16*	*β17*	*β18*	*β19*	*β20*	*β21*	*β22*	*β23*	*β24*	*β25*
1	*4TM*	*1EK9A*	+	+	+																						
2	*4TM*	*2GR8A*	+	+	+																						
3	*4TM*	*3X2RA*	+	+	+																						
4	*8TM*	*1P4TA*	+	+	+	+	-	+	+																		
5	*8TM*	*1QJPA*	+	+	+	-	+	+	+																		
6	*8TM*	*2ERVB*	+	+	-	-	-	+	+																		
7	*8TM*	*2X27X*	+	+	+	-	+	+	+																		
8	*8TM*	*3DZMA*	+	+	+	-	-	+	-																		
9	*8TM*	*3GP6A*	+	+	+	+	-	+	+																		
10	*10TM*	*2VDFA*	+	+	+	+	+	-	+	+	+																
11	*10TM*	*2X55A*	+	+	+	+	+	+	+	+	+																
12	*12TM*	*1QD6C*	+	+	+	+	+	+	+	+	+	+	+														
13	*12TM*	*1TLYA*	+	+	+	+	+	+	+	+	+	+	+														
14	*12TM*	*1UYNX*	+	+	+	+	+	+	+	+	+	+	+														
15	*12TM*	*2WJRA*	+	+	+	+	+	+	+	+	+	+	+														
16	*12TM*	*3FIDA*	+	+	+	+	+	+	+	+	+	+	+														
17	*12TM*	*4RL8A*	+	+	+	+	+	+	+	+	+	+	+														
18	*14TM*	*2X9KA*	+	+	+	+	+	+	+	+	+	+	+	+	+												
19	*14TM*	*3BS0A*	+	+	+	+	+	+	+	+	+	+	+	+	+												
20	*16TM*	*2FGQX*	+	+	+	+	-	+	+	+	+	-	+	+	+	+	+										
21	*16TM*	*4C00A*	+	+	+	+	+	+	+	+	+	-	+	+	+	+	+										
22	*16TM*	*4Y25A*	+	+	+	+	+	+	+	+	+	+	+	+	+	+	+										
23	*18TM*	*1A0TP*	+	+	+	+	+	-	+	+	+	+	+	+	+	+	+	+	-								
24	*18TM*	*2YNKA*	+	-	+	-	+	+	+	-	+	-	+	+	+	+	+	+	-								
25	*18TM*	*3SZVA*	+	+	+	+	+	-	+	+	+	+	+	-	+	+	+	-	-								
26	*19TM*	*4C69X*	+	+	+	+	+	+	+	+	+	+	+	+	+	+	+	-	-	+							
27	*22TM*	*1FEPA*	-	+	+	+	+	+	+	+	+	+	+	-	+	+	+	+	-	+	+	-	-				
28	*24TM*	*3FIPA*	+	+	+	+	+	+	+	+	+	+	+	+	-	-	-	-	-	+	+	-	-	-	-		
29	*26TM*	*4Q35A*	+	+	+	+	-	-	-	+	+	+	+	+	+	+	+	+	-	-	+	+	-	-	-	+	-

Table S6: Converted signs of γ type dihedral angles for the 29 β-barrels^{TM} proteins.

Sl#	*TM#*	*PDB*	*γ1*	*γ2*	*γ3*	*γ4*	*γ5*	*γ6*	*γ7*	*γ8*	*γ9*	*γ10*	*γ11*	*γ12*	*γ13*	*γ14*	*γ15*	*γ16*	*γ17*	*γ18*	*γ19*	*γ20*	*γ21*	*γ22*	*γ23*	*γ24*
1	*4TM*	*1EK9A*	+	+																						
2	*4TM*	*2GR8A*	+	+																						
3	*4TM*	*3X2RA*	+	+																						
4	*8TM*	*1P4TA*	+	+	+	+	-	+																		
5	*8TM*	*1QJPA*	+	+	-	+	-	+																		
6	*8TM*	*2ERVB*	+	+	-	-	-	+																		
7	*8TM*	*2X27X*	+	+	+	+	-	+																		
8	*8TM*	*3DZMA*	+	+	+	+	-	+																		
9	*8TM*	*3GP6A*	+	+	-	+	-	-																		
10	*10TM*	*2VDFA*	+	+	+	+	+	+	+	+																
11	*10TM*	*2X55A*	+	+	+	+	+	+	+	+																
12	*12TM*	*1QD6C*	+	+	+	+	+	+	+	+	+	+														
13	*12TM*	*1TLYA*	+	-	+	+	+	+	+	+	+	+														
14	*12TM*	*1UYNX*	+	+	+	+	+	+	-	+	+	+														
15	*12TM*	*2WJRA*	+	+	+	+	+	+	+	+	+	+														
16	*12TM*	*3FIDA*	+	+	+	+	+	+	+	+	+	+														
17	*12TM*	*4RL8A*	+	+	+	+	+	+	+	+	+	+														
18	*14TM*	*2X9KA*	+	+	+	+	+	+	+	+	+	+	+	+												
19	*14TM*	*3BS0A*	+	+	+	+	+	+	+	+	+	+	+	+												
20	*16TM*	*2FGQX*	+	-	-	+	+	-	+	+	+	-	+	+	+	+										
21	*16TM*	*4C00A*	+	+	+	+	+	+	+	-	+	+	+	+	+	+										
22	*16TM*	*4Y25A*	+	+	+	+	+	+	+	-	+	+	+	+	+	+										
23	*18TM*	*1A0TP*	+	+	+	+	+	+	+	+	+	+	+	-	+	+	+	+								
24	*18TM*	*2YNKA*	-	-	+	+	+	+	+	+	+	+	+	+	+	+	+	+								
25	*18TM*	*3SZVA*	+	+	+	+	+	-	+	+	+	+	+	-	+	-	+	-								
26	*19TM*	*4C69X*	-	+	+	+	+	+	+	+	+	+	+	+	+	+	+	+	+							
27	*22TM*	*1FEPA*	+	+	+	+	+	+	+	+	+	+	+	+	+	+	+	+	-	-	+	+				
28	*24TM*	*3FIPA*	+	+	+	+	+	-	+	+	+	+	+	-	-	-	+	+	-	+	+	+	-	+		
29	*26TM*	*4Q35A*	+	+	+	+	-	-	-	+	+	+	+	+	+	+	+	+	+	+	-	-	-	+	-	-

Table S7: Converted signs of β type dihedral angles for the 51 β-barrels^{cytoplasm} proteins.

SI#	TM#	PDB	β1	β2	β3	β4	β5	β6	β7	β8	β9	β10	β11	β12	β13
1	4N	2B97A	+	-	+										
2	4N	1G3PA	+	+	+										
3	5N	1NB9A	-	-	-	-									
4	5N	1WHIA	+	-	+	+									
5	5N	1PV4A	+	+	+	+									
6	5N	1GCPA	+	+	+	-									
7	5N	1HK9A	+	+	+	-									
8	5N	1R6JA	-	+	+	-									
9	5N	1O6AA	+	+	+	+									
10	5N	1G31A	+	+	-	-									
11	6N	2JDID	+	+	+	+	+								
12	6N	1WJXA	+	+	+	+	-								
13	6N	1AGJA	+	+	+	+	+								
14	6N	1DFUP	-	+	+	+	+								
15	6N	1S98A	+	-	+	+	+								
16	6N	1EFTA	+	+	+	+	+								
17	6N	2D9RA	+	+	+	+	+								
18	6N	2IMLA	-	+	+	+	+								
19	6N	1BCOA	+	+	+	+	+								
20	6N	1TS9A	+	+	+	-	+								
21	6N	2BLNA	+	+	+	+	+								
22	6N	2F1LA	+	+	+	-	+								
23	7N	1WPOA	+	+	+	+	+	-							
24	7N	1IK9A	+	+	+	+	+	+							
25	7N	1ORUA	+	-	+	+	+	+							
26	7N	1JEYA	+	+	-	+	+	+							
27	7N	1RQPA	+	+	+	-	+	+							
28	7N	1O70A	+	+	+	+	+	+							
29	7N	1J0WA	+	+	+	-	+	+							
30	7N	2GUJA	+	+	+	+	+	+							
31	7N	1QZ8A	-	+	+	+	-	+							
32	8N	1EAR	+	+	+	+	+	+	-						
33	8N	1UE0A	+	+	+	+	-	-	+						
34	8N	1A1XA	+	+	+	-	+	+	+						
35	8N	1NYCA	+	+	-	+	+	+	+						

36	8N	1OEWA	+	+	+	-	+	-	+						
37	8N	1XE1A	+	+	+	+	+	-	+						
38	8N	1T2WA	+	-	+	+	+	-	+						
39	8N	1PQHA	+	+	+	+	+	-	+						
40	8N	2CPLA	-	+	+	-	+	-	+						
41	8N	1Y12A	+	+	+	+	+	+	-						
42	8N	2Q03A	-	+	+	+	+	+	+						
43	8N	2F9HA	+	-	+	+	+	-	+						
44	8N	1BEBA	+	+	+	+	+	+	+						
45	8N	1GQBA	+	-	-	+	+	+	+						
46	8N	1F3UB	-	+	-	+	-	+	-						
47	10N	2P12A	+	+	-	+	+	+	-	-	+				
48	11N	2FR2A	+	-	+	+	+	+	-	+	+	+			
49	12N	4FGFA	+	+	-	+	+	+	-	+	+	+	-		
50	13N	1NLSA	+	+	+	+	+	+	+	+	-	+	+	+	
51	14N	1H4GA	+	-	+	+	+	+	+	-	+	-	+	+	+

Table S8: Converted signs of γ type dihedral angles for the 51 β-barrels[cytoplasm] proteins.

SI#	TM#	PDB	γ1	γ2	γ3	γ4	γ5	γ6	γ7	γ8	γ9	γ10	γ11	γ12
1	*4N*	*2B97A*	-	+										
2	*4N*	*1G3PA*	+	+										
3	*5N*	*1NB9A*	+	-	+									
4	*5N*	*1WHIA*	+	+	+									
5	*5N*	*1PV4A*	-	+	-									
6	*5N*	*1GCPA*	+	+	+									
7	*5N*	*1HK9A*	+	+	+									
8	*5N*	*1R6JA*	+	+	+									
9	*5N*	*1O6AA*	-	+	-									
10	*5N*	*1G31A*	+	-	-									
11	*6N*	*2JDID*	-	-	+	+								
12	*6N*	*1WJX*	-	+	-	-								
13	*6N*	*1AGJ*	+	-	-	-								
14	*6N*	*1DFUP*	-	+	-	-								
15	*6N*	*1S98A*	-	-	+	+								
16	*6N*	*1EFTA*	+	-	+	+								
17	*6N*	*2D9RA*	+	-	+	+								
18	*6N*	*2IMLA*	-	-	-	+								
19	*6N*	*1BCOA*	+	-	+	+								
20	*6N*	*1TS9A*	+	+	+	+								
21	*6N*	*2BLNA*	+	-	+	-								
22	*6N*	*2F1LA*	+	+	+	+								
23	*7N*	*1WPOA*	-	-	+	-	-							
24	*7N*	*1IK9A*	+	+	+	+	+							
25	*7N*	*1ORUA*	-	+	-	+	-							
26	*7N*	*1JEYA*	-	+	-	-	-							
27	*7N*	*1PJ7A*	+	+	-	+	+							
28	*7N*	*1RQRA*	+	-	+	-	-							
29	*7N*	*1J0WA*	+	+	+	+	+							
30	*7N*	*2GUJA*	+	-	+	+	-							
31	*7N*	*1QZ8A*	-	+	-	-	-							
32	*8N*	*1EARA*	-	+	-	+	-	-						
33	*8N*	*1UE0A*	+	-	-	-	+	+						
34	*8N*	*1A1X*	+	-	-	+	+	-						

35	*8N*	*1NYCA*	-	+	+	+	+	+						
36	*8N*	*1OEWA*	+	+	+	-	-	+						
37	*8N*	*1XE1A*	+	-	-	+	-	-						
38	*8N*	*1T2WA*	+	+	-	-	+	-						
39	*8N*	*1PQHA*	-	-	+	-	-	+						
40	*8N*	*2CPLA*	-	+	-	-	-	+						
41	*8N*	*1Y12A*	-	-	+	+	-	-						
42	*8N*	*2Q03A*	+	+	+	-	+	+						
43	*8N*	*2F9HA*	-	+	+	+	+	+						
44	*8N*	*1BEBA*	+	+	-	+	+	+						
45	*8N*	*1GQBA*	+	-	-	+	+	-						
46	*8N*	*1F3UB*	+	+	-	-	+	+						
47	*10N*	*2P12A*	+	+	+	+	+	+	-	+				
48	*11N*	*2FR2A*	+	+	+	+	+	-	-	+	+			
49	*12N*	*4FGFA*	+	-	-	+	+	-	+	+	+	-		
50	*13N*	*1NLSA*	+	+	-	+	-	-	-	+	+	+	-	
51	*14N*	*1H4GA*	+	+	-	-	-	-	+	-	-	+	-	-

Here, PDB codes of 29 and 51 non-homologous structures of both β-barrels^{TM} and β-barrels^{cytoplasm} proteins are given with proteins names and classifications.

Table S9: All 29 β-barrels^{TM} proteins PDB IDs, Protein names and their classification used in this work.

SI#	*TM#*	*PDB*	*Protein*	*Family*	*Superfamily*
1	***4TM***	***1EK9A***	***Outer membrane protein***	***Outer membrane efflux proteins***	***Outer membrane efflux proteins***
2	***4TM***	***2GR8A***	***Autotransporters Hia***	***Autotransporter-2 (AT-2)***	***Trimeric Autotransporters***
3	***4TM***	***3X2RA***	***Bacterial amyloid secretion channel***	***Curli-like transporters***	***Curli-like transporters***
4	***8TM***	***1P4TA***	***Outer membrane protein NspA***	***Opacity porins***	***Opacity porins***
5	***8TM***	***1QJPA***	***Outer membrane protein A (OMPA)***	***OmpA family***	***OmpA-OmpF porin family***
6	***8TM***	***2ERVA***	***Lipid A deacylase PagL***	***Lipid A 3-O-deacylase (PagL)***	***Lipid A 3-O-deacylase (PagL)***
7	***8TM***	***2X27X***	***Outer membrane protein OprG***	***OMP W (OmpW)***	***OMP W (OmpW)***
8	***8TM***	***3DZMA***	***Major outer membrane protein TtoA***	***Major OMP***	***Major OMP***
9	***8TM***	***3GP6A***	***Lipid A acylase PagP***	***Lipid A acylation***	***Lipid A acylation***
10	***10TM***	***2VDFA***	***Outer membrane adhesion/invasion OpcA***	***OM adhesion protein OpcA***	***OM adhesion***
11	***10TM***	***2X55A***	***Plasminogen activator PLA***	***OM protease omptin, OMPT***	***Omptin***
12	***12TM***	***1QD6C***	***Outer membrane phospholipase A***	***OM phospholipase A, OmpLA***	***OM phospholipase***
13	***12TM***	***1TLYA***	***Bacterial Nucleoside Transporter Tsx***	***Nucleoside transporter Tsx***	***Nucleoside-specific channel-porin (Tsx)***
14	***12TM***	***1UYNX***	***Autotransporters NalP***	***Autotransporters of N-terminal passenger***	***Autotransporters (AT)***
15	***12TM***	***2WJRA***	***Acidic sugar-specific porin***	***Oligogalactoronate-specific porin (KdgM)***	***Oligogalactoronate-specific porin (KdgM)***
16	***12TM***	***3FIDA***	***Lipid A deacylase LpxR***	***Lipid A deacylase***	***Lipid A deacylase***
17	***12TM***	***4RL8A***	***Phenol degradation pathway involved protein***	***Porin/alpha-amylase***	***Porin/alpha-amylase***
18	***14TM***	***2X9KA***	***Outer membrane protein G (OMPG)***	***OmpG porin***	***OmpG-like porins***
19	***14TM***	***3BS0A***	***Toluene transporter TodX***	***Fatty acid transporter FadL family***	***FadL outer membrane protein (FadL)***
20	***16TM***	***2FGQX***	***Anion-selective porin***	***General Bacterial Porin (GBP)***	***Trimeric porins***
21	***16TM***	***4C00A***	***Translocation and assembly module TamA***	***OMP insertion (Bam complex) porin***	***Omp85-TpsB transporters***
22	***16TM***	***4Y25A***	***Poly-beta-1,6-N-acetyl-D-glucosamine export***	***Poly acetyl glucosamine porin***	***Poly acetyl glucosamine porin***
23	***18TM***	***1A0TP***	***Sucrose-specific porin***	***Malptoporin-like proteins***	***Sugar porins***
24	***18TM***	***2YNKA***	***Capsule assembly protein***	***Capsule assembly protein Wzi***	***Capsule assembly protein***
25	***18TM***	***3SZVA***	***Pyroglutamate porin***	***Outer membrane porin (Opr)***	***OprD/AlgE superfamily***
26	***19TM***	***4C69X***	***VDAC-1 channel***	***Voltage-dependent anion channel (VDAC)***	***Mitochondrial and plastid porins***
27	***22TM***	***1FEPA***	***Ferric enterobactin receptor***	***Outer Membrane Receptor (OMR)***	***Ligand-gated protein channels***
28	***24TM***	***3FIPA***	***P pilus usher PapC translocation domain***	***Fimbrial usher porin***	***Fimbrial usher porin***
29	***26TM***	***4Q35A***	***LPS-assembly protein complex***	***LPS assembly protein family***	***LPS assembly protein family***

Table S10: All 51 β-barrels^{cytoplasm} proteins PDB IDs, Protein names and their classification used in this work.

SI#	N #	PDB	Protein names	Protein state
1	*4N*	*2B97A*	*Hydrophobin II, HfbII*	*Closed*
2	*4N*	*1G3PA*	*N-terminal domains of the minor coat protein g3p*	*Opened*
3	*5N*	*1NB9A*	*Reductase/isomerase/elongation factor common domain*	*Closed*
4	*5N*	*1WHIA*	*Ribosomal protein L14*	*Closed*
5	*5N*	*1PV4A*	*OB-fold*	*Opened*
6	*5N*	*1GCPA*	*SH3-like barrel*	*Opened*
7	*5N*	*1HK9A*	*Sm-like fold*	*Opened*
8	*5N*	*1R6JA*	*PDZ domain-like*	*Opened*
9	*5N*	*1O6AA*	*Surface presentation of antigens (SPOA)*	*Opened*
10	*5N*	*1G31A*	*GroES-like*	*Opened*
11	*6N*	*2JDID*	*Domain of alpha and beta subunits of F1 ATP synthase-like*	*Closed*
12	*6N*	*1WJXA*	*Small protein B (SmpB)*	*Closed*
13	*6N*	*1AGJA*	*Trypsin-like serine proteases*	*Closed*
14	*6N*	*1DFUP*	*Ribosomal protein L25-like*	*Closed*
15	*6N*	*1S98A*	*HesB-like domain*	*Closed*
16	*6N*	*1EFTA*	*Elongation factor/amino methyltransferase*	*Closed*
17	*6N*	*2D9RA*	*Double-split beta-barrel*	*Closed*
18	*6N*	*2IMLA*	*Split barrel-like*	*Opened*
19	*6N*	*1BCOA*	*mu transposase, C-terminal domain*	*Opened*
20	*6N*	*1TS9A*	*Rof/RNase P subunit-like*	*Opened*
21	*6N*	*2BLNA*	*FMT C-terminal domain-like*	*Opened*
22	*6N*	*2F1LA*	*PRC-barrel domain*	*Opened*
23	*7N*	*1WPOA*	*Herpes virus serine proteinase, assemblin*	*Closed*
24	*7N*	*1IK9A*	*XRCC4, N-terminal domain*	*Closed*
25	*7N*	*1ORUA*	*PK beta-barrel domain-like*	*Closed*
26	*7N*	*1JEYA*	*SPOC domain-like*	*Closed*
27	*7N*	*1RQPA*	*Bacterial fluorinating enzyme, C-terminal domain*	*Closed*
28	*7N*	*1O70A*	*FAS1 domain*	*Closed*
29	*7N*	*1J0WA*	*PH domain-like barrel*	*Opened*
30	*7N*	*2GUJA*	*Phage tail proteins*	*Opened*
31	*7N*	*1QZ8A*	*Replicase NSP9*	*Opened*

32	*8N*	*1EARA*	*Urease metallochaperone UreE*	*Closed*
33	*8N*	*1UE0A*	*ValRS/IleRS/LeuRS editing domain*	*Closed*
34	*8N*	*1A1XA*	*Oncogene products*	*Closed*
35	*8N*	*1NYCA*	*Streptavidin-like*	*Closed*
36	*8N*	*1OEWA*	*Acid proteases*	*Closed*
37	*8N*	*1XE1A*	*Reductase/isomerase/elongation factor common domain*	*Closed*
38	*8N*	*1T2WA*	*Sortase*	*Closed*
39	*8N*	*1PQHA*	*Double psi beta-barrel*	*Closed*
40	*8N*	*2CPLA*	*Cyclophilin-like*	*Closed*
41	*8N*	*1Y12A*	*Hcp1-like*	*Closed*
42	*8N*	*2Q03A*	*AOC barrel-like*	*Closed*
43	*8N*	*2F9HA*	*PTSIIA/GutA-like*	*Closed*
44	*8N*	*1BEBA*	*Lipocalins*	*Opened*
45	*8N*	*1GQBA*	*Mannose 6-phosphate receptor domain*	*Opened*
46	*8N*	*1F3UB*	*Triple barrel*	*Opened*
47	*10N*	*2P12A*	*FomD barrel-like*	*Closed*
48	*11N*	*2FR2A*	*Lipocalins*	*Opened*
49	*12N*	*4FGFA*	*beta-Trefoil*	*Closed*
50	*13N*	*1NLSA*	*Concanavalin A-like lectins/glucanases*	*Closed*
51	*14N*	*1H4GA*	*Concanavalin A-like lectins/glucanases*	*Closed*
